# Infection of Monocytes From Tuberculosis Patients With Two Virulent Clinical Isolates of *Mycobacterium tuberculosis* Induces Alterations in Myeloid Effector Functions

**DOI:** 10.3389/fcimb.2020.00163

**Published:** 2020-04-23

**Authors:** Lelia Lavalett, Hector Ortega, Luis F. Barrera

**Affiliations:** ^1^Grupo de Inmunología Celular e Inmunogenética (GICIG), Facultad de Medicina, Universidad de Antioquia, Medellín, Colombia; ^2^Facultad de Ciencias, Universidad Nacional de Colombia Sede Medellín, Medellín, Colombia; ^3^Clínica Cardiovascular Santa María, Medellín, Colombia

**Keywords:** human, monocytes, patients, *M. tuberculosis*, clinical isolates, microarray, transcriptome

## Abstract

Monocytes play a critical role during infection with *Mycobacterium tuberculosis* (Mtb). They are recruited to the lung, where they participate in the control of infection during active tuberculosis (TB). Alternatively, inflammatory monocytes may participate in inflammation or serve as niches for Mtb infection. Monocytes response to infection may vary depending on the particularities of the clinical isolate of Mtb from which they are infected. In this pilot study, we have examined the baseline mRNA profiles of circulating human monocytes from patients with active TB (MoTB) compared with monocytes from healthy individuals (MoCT). Circulating MoTB displayed a pro-inflammatory transcriptome characterized by increased gene expression of genes associated with cytokines, monocytopoiesis, and down-regulation of MHC class II gene expression. In response to *in vitro* infection with two clinical isolates of the LAM family of Mtb (UT127 and UT205), MoTB displayed an attenuated inflammatory mRNA profile associated with down-regulation the *TREM1* signaling pathway. Furthermore, the gene expression signature induced by Mtb UT205 clinical strain was characterized by the enrichment of genes in pathways and biological processes mainly associated with a signature of IFN-inducible genes and the inhibition of cell death mechanisms compared to MoTB-127, which could favor the establishment and survival of Mtb within the monocytes. These results suggest that circulating MoTB have an altered transcriptome that upon infection with Mtb may help to maintain chronic inflammation and infection. Moreover, this functional abnormality of monocytes may also depend on potential differences in virulence of circulating clinical strains of Mtb.

## Introduction

*Mycobacterium tuberculosis* (Mtb) is an intracellular pathogen preferentially infecting cells of the myeloid lineage, including macrophages and monocytes. Although the immunological response to Mtb infection has been intensively explored, the early interactions of innate immune cells and Mtb are limited, particularly in the human infection (Sia et al., 2015). The innate immune system can clear the infection of Mtb before the initiation of an adaptive immune response. However, Mtb can disturb different processes within the host cells to altering particular signaling pathways and preventing the activities of specific bactericidal mechanisms (Flannagan et al., 2009; Lerner et al., 2015).

Monocytes have been described as playing a dual role during Mtb infection. They are essential in the anti-mycobacterial defense through innate activation mechanisms and triggering the acquired immunity against Mtb; however, they may also participate in pathogen dissemination, inflammatory reaction, and the tissue damage occurring during TB (Pahari et al., 2018). It has reported that circulating monocytes from patients with pulmonary tuberculosis (TB) exhibit phenotypic and functional alterations compared with healthy controls (Sanchez et al., 2006; Castaño et al., 2011a; Chavez-Galan et al., 2015), which can be associated with disease progression or severity. However, the transcriptional response that could be associated with such alterations in monocytes from TB patients is unknown.

The transcriptome of human monocytes and monocyte subsets has been examined under different circumstances, mostly from healthy control individuals (MoCT) (Martinez et al., 2006; Ancuta et al., 2009; Fairfax et al., 2014; Rinchai et al., 2016; Kapellos et al., 2019). However, much less information exists concerning the transcriptome of human monocytes from TB patients (MoTB) (Esterhuyse et al., 2015; Sampath et al., 2018), and no actual information has been reported on the consequences of Mtb infection of circulating MoTB based on the potential consequences of monocytes infection at the sites of infection. Using genome-wide global gene expression, we first investigated transcriptional responses of circulating MoTB, aiming to understand the systemic effect of the disease on its gene expression profile. Since monocytes are recruited to the infection foci where they encounter with Mtb (Antonelli et al., 2010; Huang et al., 2018), we then analyzed the mRNA profile response of MoTB *in vitro* infected with two Mtb clinical strains (UT127 and UT205) and compared this response to the profiles of MoCT. We found that the baseline transcriptome of circulating MoTB show an altered transcriptional profile compared to MoCT. The expression of genes associated with inflammatory response, cellular chemotaxis, monocytopoiesis, TNF, and HMGB1 signaling pathways were significantly increased in MoTB. Also, MoTB negatively regulated the expression of genes encoding MHC II molecules such as *CD74, HLA-DRB6, HLA-DPA1, HLA-DRB3*, and *HLA-DRB4*, possibly as a systemic consequence of the active TB. Interestingly, upon *in vitro* infection with Mtb, MoTB (MoTB-127 and MoTB-205) displayed an attenuated gene expression profile compared with MoCT (MoCT-127 and MoCT-205). Infected MoTB-127 were unable to express genes associated with infection control, and displayed alterations in myeloid effector function associated with differentiation, endocytosis, phagosome maturation, and the TREM1 signaling pathway. Interestingly, MoTB-205 showed a strong signature of IFN-inducible genes and the inhibition of cell death mechanisms compared to MoTB-127.

These findings provide insight into a combination of host factors and Mtb virulence factors that can influence multiple aspects of the host immune response during active TB, which could define the gene expression profile seen in MoTB.

## Materials and Methods

### Subjects

Healthy volunteers (*n* = 4), 2 females (mean age 25, range 25) and 2 males (mean age 26.5 range 24–29), and patients with active pulmonary TB (*n* = 4), 2 females (mean age 33.5, range 22–45) and 2 males (mean age 23 range 21–25), were used as a source of peripheral venous blood for obtaining monocytes, as described below. The samples from the TB patients were obtained within the first 2 weeks of treatment with antibiotic treatment for TB. All individuals were between 18 and 50 years old, non-smoking individuals, negatives for HIV, cancer, and diabetes at the time of sampling, voluntarily participated in this study.

### *Mycobacterium tuberculosis* Clinical Isolates

*Mycobacterium tuberculosis* clinical isolates UT205 and UT127 were obtained and characterized by the Centro Colombiano para la Investigación en Tuberculosis (CCITB) from a cohort study of TB patients and their household contacts (del Corral et al., 2009; Realpe et al., 2014). Both isolates belong to the LAM family, which contributes to the majority of cases of TB in the Metropolitan Area of Medellin, Colombia (Realpe et al., 2014).

The UT127 and UT205 strains were initially selected based on epidemiological evidence. While there was an incident case in the household of the TB patient infected with UT205, there were no incident cases in the household of the TB patient infected with UT127 (del Corral et al., 2009). A posterior study (Duque et al., 2014) indicated that infection of human monocytes (MOI 10:1) with UT205 for 24 h induced a higher proportion of cells with membrane damage compared to UT127, suggesting differences in virulence between these two isolates. We also sequenced the genomes of both isolates (Isaza et al., 2012; Baena et al., 2019), which are 99.6% identical. Interestingly, they display significant transcriptomic differences when growth in axenic media (Baena et al., 2019) besides their extensive genomic similarity. Also, our laboratory has reported significant differences in phagocytic activity, cytokines production, and cell death phenotype in human monocytes infected with clinical isolate UT127 compared with H37Rv (Castaño et al., 2014). Mtb clinical isolates were cultured as previously described (Rojas et al., 1997). Briefly, mycobacteria were grown in Middlebrook 7H9 broth supplemented with 10% OADC (Becton Dickinson, NY) and Tween 80 (0.05%) for 2–3 weeks to reach the exponential growth phase, and clumps disrupted by sonication (CV33 Sonics Vibra Cell, Newtown, CT). The bacterial suspension was diluted in freezing medium and frozen at −70°C until used. To determine the number of colony-forming units (CFU), 20 μl of serial dilutions were plated onto Petri dishes (Corning, NY) containing Middlebrook 7H10 agar supplemented with glycerol, and 10% OADC pH 7.2 at 37°C for 3 weeks. Upon thawing, mycobacterial viability (usually more than 90%) was tested using FDA stained bacteria by flow cytometry (Norden et al., 1995).

### Isolation of Monocytes

Peripheral blood mononuclear cells (PBMC) were used as a source for circulating monocytes. Monocyte monolayers from MoCT and MoTB were established as previously described (Castaño et al., 2011a). Briefly, defibrinated blood samples (~50 ml) were centrifuged on Histopaque (Sigma, St. Louis, MO), washed with Phosphate Saline Dulbecco (DPBS, GIBCO, Life Technologies Grand Island, NY), and the viability (≥95%) was determined by trypan blue exclusion. 5 × 10^5^ CD14^+^ cells were seeded in 24-well plates (Corning Incorporated Life Science, Lowell, MA) using 1 ml of RPMI-1640 (Invitrogen, Grand Island, NY) supplemented with 0.5% AB+ inactivated human serum (Invitrogen, Brown Deer, WI), penicillin and streptomycin (Biowittaker, Walkersville, MD) (complete medium, CM), for 4 h at 37°C, 5% CO_2_, 95% relative humidity. Cells were washed extensively to remove non-adherent cells with pre-warmed (37°C) DPBS supplemented with 0.5% AB+ inactivated human serum. In these conditions, CD14^+^ cells represented >95% of the adherent cells (results not shown).

### Experimental Study Design

We first analyze the systemic effect of the disease on the basal profiles of MoTB gene expression compared to MoCT (Supplementary Figure 1). For this, adherent cells were lysed in buffer RLT (Qiagen) with 1% β-mercaptoethanol and stored at −80°C until use for RNA extraction. Second, we analyzed the effect of *in vitro* infection with the clinical isolates of Mtb UT127 and UT205 in MoTB and MoCT (Supplementary Figure 1). Monocytes were infected for 6 h with Mtb at a multiplicity of infection (MOI) of 10:1. The cells were extensively washed with pre-warmed (37°C) DPBS supplemented with 0.5% AB+ inactivated human serum to eliminate non-ingested bacteria. Then, adherent cell were lysed in buffer RLT (Qiagen) with 1% β-mercaptoethanol and stored at −80°C until use for RNA extraction. Three experimental settings were evaluated, including (1) *In vitro* non-infected samples (NI) used as a control, (2) samples *in vitro* infected with Mtb UT127, and (3) samples *in vitro* infected with Mtb UT205 (Supplementary Figure 1). The percentage of infected monocytes in the experimental conditions used in this study (MOI 10:1) was defined in preliminary experiments using the Kinyoun staining. At least 400 cells in randomly selected fields were counted. In all cases, the proportion of infected macrophages was superior to 85% (data not shown).

### RNA Isolation

The whole procedure for total RNA isolation was previously described (Lavalett et al., 2017). Essentially, total RNA was obtained from 3 to 5 replica wells. Total RNA was collected by RNA spin columns (RNeasy Plus Mini Kit, Qiagen, Carlsbad, Germany) according to the manufacturer's protocol. The quality of the RNA was assessed by measuring the ratio of absorbance at 260 and 280 nm using a Nanodrop 2000 Spectrometer (Thermo Scientific), and the integrity of the RNA (RIN) was assessed using the Agilent 2100 Bioanalyzer (Agilent Technologies, Waldbronn, Germany). Only samples with a RIN > 7 were considered (Average median RIN = 9.2; IQ = 0.8; range = 7.5–9.9). Total RNA from all samples was prepared simultaneously to reduce the sample to sample variability.

### Microarray Analyses and Preprocessing of Data

All conditions for microarray analysis were performed as previously described (Lavalett et al., 2017). Seven hundred and fifty nanograms of labeled cRNA samples were hybridized to each Illumina Human BeadChip (Illumina, San Diego, CA) HT-12 v.4.0 bead array, and samples processed according to the manufacturer's instructions. This BeadChip provides coverage of 47,231 probes targeting more than 31,000 annotated genes, from which 22,283 are unique. Raw data were extracted by the Illumina GenomeStudio v2011.1 (Gene Expression Module v1.9.0). All data have been submitted to the NCBI gene expression omnibus (GEO) database accession number GSE139871.

The raw data were preprocessed by the Lumi Bioconductor package (Du et al., 2008), using the R software. Computational analyses with the Lumi package, including a variance stabilizing transformation (Lin et al., 2008) and quantile normalization steps. Differentially expressed genes (DEGs) were obtained using the Limma package (Smyth, 2004). Criteria for the selection of DEGs included a log2 fold change (FC ≥ 1.5 or ≤1.5), *P*-value (<0.05), and False Discovery Rate (FDR) value (<0.05) (Supplementary Figure 1).

### Functional Enrichment and Canonical Pathway Analyses

Functional enrichment analysis was performed as previously described (Lavalett et al., 2017), using Gene Ontology (GO), the Kyoto Encyclopedia of Genes and Genomes (KEGG) Pathways (Kanehisa et al., 2004), the Innate database (InnateDB) (Breuer et al., 2013), and STRING database to do functional association analysis (Szklarczyk et al., 2017) (Supplementary Figure 1). IPA (Ingenuity Pathway Analysis) was used to classify DEGs into functional relationships, and to show canonical pathways and networks involving these genes with potentially critical host mediators of TB disease. IPA ranks the pathways according to the enrichment score (Fisher's exact test *P*-value) and the Z-score. Activation (Z-score ≥ 2) or inhibition (Z-score ≤ 2) of the pathways is predicted by comparing observed and predicted regulation patterns.

### Technical Validation of Differential Expression by Quantitative Real-Time PCR

Validation and confirmation of microarray results were performed by qRT-PCR as described (Lavalett et al., 2017). Nine genes (*IL1B, CXCL10, PTGS2, TNF, TNFAIP6, SERPINB2, CCL20, IL6, and IL8*) were selected as those with the highest values of expression in the lists of DEGs as well as for their possible functions in TB. The RNA samples used for qRT-PCR were the same as those used for the microarray (samples from three individuals from each group including RNAs in duplicate of the three experimental settings NI, samples infected with Mtb UT127 and infected with Mtb UT205). In summary, 100 ng of total RNA was reverse transcribed, and the PCR products quantified using the Rotor-Gene Q (Qiagen, Carlsbad, Germany) and the Platinum® SYBR® Green qPCR SuperMix was used to produce fluorescent-labeled PCR products according to the manufacturer's instructions. Published and validated primers sets (Zhang et al., 1995, 2014; Coldren et al., 2003; Volpe et al., 2006; Bakshi et al., 2008; Williams et al., 2011; Tomlinson et al., 2012; Hsu et al., 2013; Reyes et al., 2015) were used (Supplementary Table 1). The relative gene expression was calculated using REST (Relative Expression Software Tool V2.0.21) (Pfaffl et al., 2002), using β-Actin (ACTB) as a normalizer gene (Ishii et al., 2006). The relative expression values obtained by qRT-PCR were transformed to log_2_FC and compared with the log_2_FC expression values obtained by the microarray method for the genes selected, and graphs were plotted using GraphPad Prism (v. 6.0).

## Results

Filtering was applied to select transcripts significantly detected (*p* < 0.05) from the background. This filtering left 20,037 out of 47,237 transcripts deposited on the Illumina chips. Transcriptomes of non-infected monocytes from control subjects (MoCT-NI) clustered from transcriptomes of non-infected monocytes from TB patients (MoTB-NI). Furthermore, transcriptomes of non-infected MoCT and MoTB clustered apart from transcriptomes of infected MoCT and MoTB, although no apparent differences were observed among them (Supplementary Figure 2). Thus, circulating MoTB seem to express a different transcriptome compared to MoCT, and infection with Mtb induces a notable transcriptomic change compared to non-infected monocytes.

To investigate the biological pathways and critical processes in the pathogenesis of TB potentially mediated by circulating monocytes, we first analyzed the systemic effect of TB on the baseline genome-wide gene expression profiles of MoTB compared to MoCT. Second, MoCT and MoTB were infected for 6 h at a multiplicity of infection of 10:1 with two Colombian clinical isolates of Mtb, UT127 and UT205, that may differ in virulence (Isaza et al., 2012; Duque et al., 2014; Baena et al., 2019), and then their transcriptional profiles were investigated.

### Analysis of Circulating Monocytes From TB Patients: Systemic Effect of the Disease

**Figure 1:**
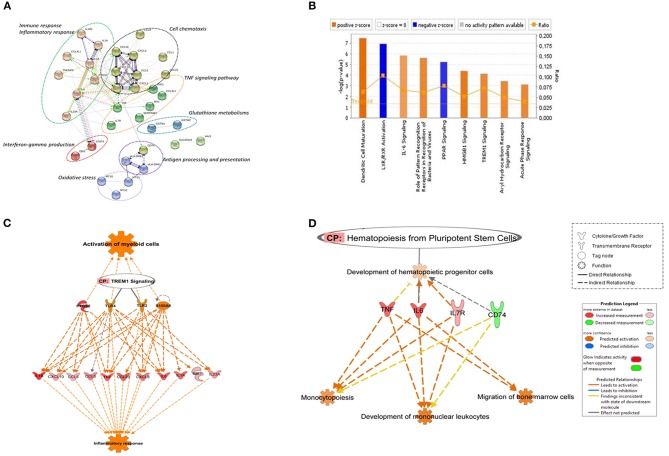
Canonical pathways based on core DEGs in CD14+ monocytes from TB individuals vs. monocytes from healthy individuals. **(A)** The STRING functional association network was generated using the list of 48 DEGs in MoTB compared to MoCT. The network nodes represent the proteins encoded by DEGs. Different colored lines connecting the nodes represent the types of evidence used in predicting associations. The graphs encircled with ovals display different groups of genes correlated with their functional categories by the biological process (GO) and KEGG pathways **(B)**. Significantly enriched canonical pathways were identified. Blue color indicates a negative z-score (≤ −2) and down-regulation of the pathway and orange color indicates a positive z-score (≥ 2) and up-regulation of the pathway. Ratio denotes the number of significantly expressed genes compared with the total number of genes associated with the canonical pathway. The orange line stands for the threshold above which there are statistically significantly (by default *P* < 0.05). **(C)** IPA prediction of upstream regulators of the list of DEGs in MoTB. PTGS2, TLR2, TLR4, and S100A8 were found as master upstream regulators that were predicted to be activated in MoTB. Canonical Pathway (CP) TREM1 was also activated. Activation of regulators and functions “Activation of myeloid cells” and “Inflammatory response” (displayed in orange) are based on IPA activation z-score ≥ 2 and *P* < 0.05. Up-regulated genes are highlighted in red, and the color depth is correlated to the fold change. Orange dashed lines with arrows indicate indirect activation. **(D)** IPA prediction of canonical pathways and biological functions of the list of DEGs in MoTB. Canonical Pathway (CP) “Hematopoiesis from pluripotent stem cell” was predicted to be activated in MoTB. Activation of functions as “Monocytopoiesis,” “Development of mononuclear leukocytes” and “Migration of bone marrow cells” (displayed in orange) were associated with TNF, IL6, IL7R, and CD74 and genes, and are based on IPA activation z-score ≥ 2 and *P* < 0.05. Up-regulated genes and down-regulated genes are highlighted in red and green, respectively. The color depth is correlated to the fold change. Orange dashed lines with arrows indicate indirect activation.

The global transcriptome of circulating MoTB was investigated, hypothesizing that these cells, compared to MoCT, could have an altered transcriptional profile as a consequence of the systemic impact of the disease during the active phase. Forty eight DEGs (35 up- and 13 down-regulated genes) were significantly regulated in MoTB relative to MoCT, indicating that specific genes are induced in response to the disease (Supplementary Figure 3, Supplementary Table 2). Enrichment analysis, which identifies specific interaction networks, was used to infer the function of these genes. Modules were grouped using their KEGG or GO annotations (FDR < 0.05) and cross-validated with the STRING database (Figure 1A). The full list of enriched gene sets is provided in Supplementary Table 3.

A detailed examination of the up-regulated DEGs expressed by MoTB highlighted many genes described as important during TB (Domingo-Gonzalez et al., 2016). These genes include cytokines such as *IL1A, IL1B, IL6, IL8, IL23A*, and *TNF*, and the chemokines *CCL1, CCL3L1, CCL4L1, CCL5, CCL8, CCL20, CXCL1, CXCL2, CXCL10*, enzymes such as *PTGS2* and *IDO1*, which were significantly associated with immune response, inflammatory response, TNF signaling pathway and cell chemotaxis (Figure 1A). These genes constituted the central core of the monocyte response to TB and are involved in the activation of immune mechanisms. Other up-regulated genes were also associated with regulation of interferon-gamma production (*EBI3* and *STAT4*), oxidative stress (Metallothionein family genes such as *MT1E, MT1G*, and *MT2A*), and glutathione metabolisms (*GSTM1* and *GSTM2*).

The top down-regulated genes included *CCL24, IL1R2*, and *VAV3*. Interestingly, MHC class II antigen processing and presentation genes, including *CD74, HLA-DRB6, HLA-DPA1, HLA-DRB3, HLA-DRB4*, were also down-regulated (Figure 1A).

To better understand the cellular and molecular functions associated with DEGs in MoTB, they were subjected to pathway analysis using QIAGEN's IngenuityPathway Analysis (IPA). Top enriched canonical pathways, including “Dendritic Cell Maturation,” “IL-6 Signaling,” “Role of Pattern Recognition Receptors in Recognition of Bacteria and Viruses,” “HMGB1 Signaling,” “TREM1 Signaling,” “Aryl Hydrocarbon Receptor Signaling” and “Acute Phase Response Signaling,” were activated in MoTB, while “LXR/RXR Activation” and “PPAR signaling” were inhibited (Figure 1B). Additionally, we performed an upstream analysis by IPA, which predicts the activation or inhibition of upstream regulators derived from the Ingenuity Knowledge Base and that explains the observed gene expression changes in our data (Figures 1C,D). This analysis revealed the activation of four molecules: *TLR2, TLR4, S100A8*, and *PTGS2*. The effect of these molecules on DEGs by MoTB shows the activation of myeloid cell functions such as *TREM1* signaling and inflammatory response (Figure 1C). Of note, the canonical pathway “Hematopoiesis from pluripotent stem cell” was also enriched in MoTB (Figure 1D, Supplementary Table 3).

Based on the reported functional and phenotypic alterations of monocytes from TB patients (Sanchez et al., 2006; Balboa et al., 2011; Castaño et al., 2011a,b; Lastrucci et al., 2015), and the transcriptomic alterations observed by us in circulating MoTB, the effects of infection with the Mtb strains UT127 and UT205 on the transcriptomes of MoCT and MoTB were examined. Thus, 120 DEGs, 103 upregulated and 17 downregulated, were observed in MoCT infected with UT127; 249 DEGs were observed in MoCT infected with UT205, 187 upregulated, and 62 downregulated. On the other hand, 143 DEGs were observed in MoTB infected with UT127, 75 upregulated, and 68 downregulated, while 79 DEGs were observed in MoTB infected with UT205, 64 upregulated and 15 downregulated (Supplementary Figure 3). A full list of DEGs for each group is provided in Supplementary Table 2.

### Common Response of MoCT and MoTB to Infection With *Mtb*

**Figure 2:**
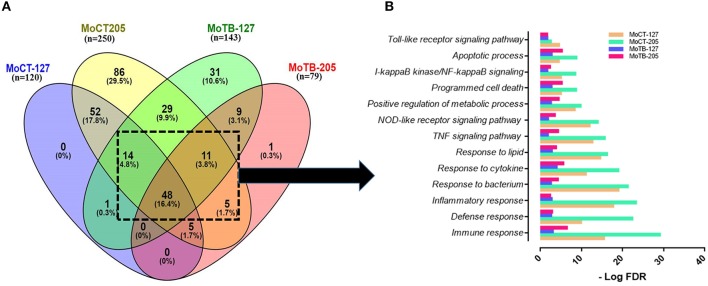
Signaling pathways and biological processes differentially affected by *in vitro* infection of monocytes from healthy vs. TB individuals. **(A)** Common DEGs (*n* = 78) expressed by monocytes (Mo) from healthy control individuals (MoCT) and TB patients (MoTB) infected with Mtb UT127 (MoCT-127, MoTB-127) or UT205 (MoCT-205, MoTB-205). **(B)** Biological response of shared DEGs by MoCT and MoTB infected with *M. tuberculosis* UT127 or UT205. GO and KEGG pathways represented were significantly enriched (FDR ≤ 0.01).

Then, we first selected the common genes expressed by MoCT in response to infection with Mtb UT127 or UT205. The same procedure was performed for MoTB. Finally, both lists were compared, and the common genes of MoCT and MoTB infected with Mtb were selected (Figure 2A, Supplementary Table 4).

The pair-wise analysis revealed a total of 78 genes that were shared in at least three or four groups (MOCT-127, MOCT-205, MOTB-127, and MOTB-205) and represent the core of global monocyte transcriptional response to Mtb infection (Figure 2A, Supplementary Table 4). The 78 common genes in monocytes infected with Mtb were subjected to functional enrichment analysis, which revealed that most of the biological processes generated by the infection are associated with the regulation of immune response, cell survival, and cellular activity (Figure 2B), a common finding in monocytes/macrophages infected with Mtb. The core gene signature consisted mainly of genes that were up-regulated (*NFKB1, BIRC3, TRAF1, STAT4, MAP3K8, RIPK2, CASP1, SERPINB9, CCL5, CCR7, IL7R, CCL14, TNFRSF4, NAMPT, SLAMF7, SOCS3, HCK, TNIP1*, and *TNIP3*), involved in the innate immune response, inflammatory response, cytokine response, positive regulation of I-kappaB kinase/NF-kappaB, TNF, NOD-like receptor and Toll-like receptor signaling pathway and regulation of apoptotic process. Additionally, metabolic processes were enriched, which consisted of nodes with up-regulated genes involved in NAD and nicotinamide nucleotide metabolic processes (*KYNU, AK4, PDE4B, CKB, ADORA2A*, and *ADA*).

In contrast, down-regulated gene interactions were also enriched and associated with regulation of lipid, regulation of carboxylic acid, and oxoacid metabolic process (*DHRS9, CKLF, LTA4H, CEBPA*, and *PPARG*). Although this response was common between MoCT and MoTB infected with UT127 or UT205, the value of significant enrichment score in signaling pathways and biological processes differs between groups, highlighting that the response to infection with both clinical isolates is decreased in MoTB compared to MoCT, particularly with UT205 (Figure 2B).

### MoCT Response to Mtb UT127 and UT205

Fifty-two genes (47 up-regulated and 5 down-regulated genes) were common MoCT when infected with UT127 and UT205 Mtb strains. No gene was exclusively expressed in response to UT127 infection, whereas 86 DEGs were unique in response to UT205 infection (Figure 2A, Supplementary Table 4). Some of the 52 common genes in MoCT in response to infection with UT127 and UT205 included pro-inflammatory cytokines and chemokines such as *IL1A, IL1B, IL6, IL23A, CCL20, TNF, TNFAIP6, CSF2, CCL1, CXCL1*, and *CXCL2* which are essential mediators of the inflammatory milieu in the lungs during TB; *PTGS2* (*COX-2*, Cyclooxygenase-2), promotes the synthesis of prostaglandin E2 (PGE2) and is crucial for bacterial control. Interestingly, this pro-inflammatory profile was similar to the baseline transcriptional profile observed in MoTB described above. However, gene expression values were higher in MoCT after infection with Mtb.

**Figure 3:**
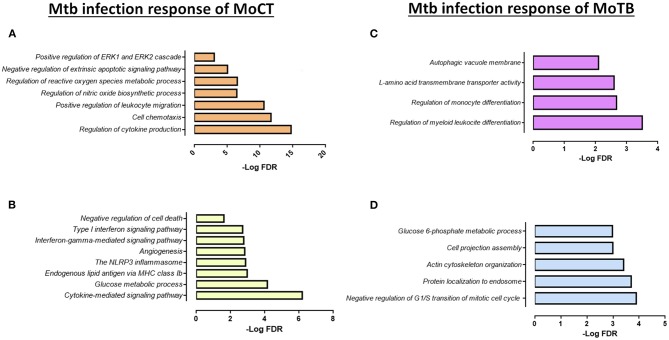
Functional categories of the biological process by GO and KEGG pathways significantly enriched (FDR ≤ 0.01) in response to infection with Mtb clinical isolates in MoCT and MoTB. **(A)** Common DEGs (*n* = 52) in MoCT infected with UT127 and UT205 clinical isolates. **(B)** Unique DEGs (*n* = 86) in MoCT In response to infection with the clinical isolate UT205. **(C)**. Unique DEGs (*n* = 9) in response to infection with Mtb UT127 and UT205. **(D)** Unique DEGs (*n* = 31) in response to infection with Mtb UT127.

Biological functions of 52 common genes to Mtb infection were mainly to the cytokine response, cellular response to molecules of bacterial origin, inflammatory response, and regulation of leukocyte migration and cell chemotaxis (Figure 3A). Additionally, significant enrichment of genes associated with the regulation of nitric oxide biosynthetic and regulation of reactive oxygen species was observed. Likewise, there was a response mainly associated with negative regulation of extrinsic apoptotic signaling pathway. A full list of enriched gene sets based on GO terms and KEGG pathways are provided as Supplementary Table 3. Of interest, the expression of three microRNAs (miRNAs) such as *MIR146A* (LOC285628), *MIR155HG* and *MIR320c* was observed, which have been reported to play a critical role in the pathogenesis of TB (Wang et al., 2014).

In response to infection with the clinical isolate UT205, 86 DEGs were unique in MoCT, in which 65 genes were up-regulated, and 21 genes were down-regulated (Figure 2A, Supplementary Table 4). The up-regulated genes were significantly associated with a strong response to cytokines and chemokines (*CCL2, CCL22, CCL23, CCL8, CXCL10*), as well as cellular response to type II and type I interferon signaling pathways (*IRF1, IFIT2, ISG20, GBP1, GBP2, GBP5, MX1, MT2A*), and negative regulation of cell death (*REL, CFLAR, TNFAIP8*) (Figure 3B). Down-regulated genes affect pathways and biological processes related to glucose metabolic process (*SDS, FABP5, HK1, PDK4*), angiogenesis and focal adhesion (*TGFBI, VAV3, VEGFB*), antigen processing and presentation, and endogenous lipid antigen via MHC class II (*CD1D*) (Figure 3B).

### MoTB Express a Distinctive Transcriptional Profile to Infection With Mtb Clinical Isolates Compared to MoCT

Although there are no reports of monocytes *in vivo* infected with Mtb in peripheral blood of patients with TB, they can be recruited to the sites in the lung where Mtb is actively replicating. As we indicated above, circulating MoTB show a pre-activated transcriptional profile compared to MoCT, associated with the systemic effect of TB due to dysregulated egress from bone marrow and/or being functionally altered in blood by entering into contact with circulating mycobacterial products. Therefore, we hypothesized that these monocytes during active TB could show an altered transcriptomic response upon infection with Mtb. Since these responses have been poorly studied, we focused our analysis by comparing the common and specific transcriptional response of MoTB infected with both clinical isolates.

Upon infection with Mtb, MoTB showed an attenuated response concerning the number of DEGs, gene expression direction (most of the genes were down-regulated), and regarding the enrichment and type of pathways and biological processes associated with the infection as compared with MoCT (Supplementary Tables 3, 4). Thus, when comparing the lists of DEGs in MoTB-127 and MoTB-205, only 9 genes were common (Figure 2A, Supplementary Table 4). Of these, 7 genes were down-regulated (*EMP1, EGR2, SGK223, CCND2, FOS, SPRY2, DCSTAMP*) and associated with negative regulation of biological processes such as myeloid leukocyte differentiation, monocyte differentiation, cell proliferation, and cell growth, while two up-regulated genes (*MAP1LC3A* and *SLC43A2*) were associated with biological processes of regulation of autophagy and L-amino acid transmembrane transporter activity, respectively (Figure 3C, Supplementary Table 3).

In order to define whether MoTB could respond differentially to different clinical strains, we examined the response to MoTB to infection with UT127 and UT205, individually. In response to infection with UT127, 31 genes were differentially expressed by MoTB, of which 7 genes were up-regulated, and 24 genes were down-regulated (Figure 2A, Supplementary Table 4). Interestingly, these genes were not associated with innate immune response functions as observed in MoCT in response to infection with Mtb, but instead were associated with the negative regulation of cellular metabolism processes such as regulation of G1/S transition of mitotic cell cycle (*FHL1, SLFN11, ATP2B4, RDX*), protein localization to endosome (*PACSIN2, SORT1, SLC30A3, RDX*), actin cytoskeleton organization (*PACSIN2, CSF1R, TACSTD2, RDX, CORO2A, NCK2*), cell projection assembly (*TBC1D10C, TACSTD2, RDX, VCL, NCK2*), and glucose 6-phosphate metabolic process (*SHPK, HK3*) (Figure 3D, Supplementary Table 3). Only one gene (*GREM1*) was unique in response to infection with UT205 and regulated negatively. Although the function of *GREM1* is unknown in TB, it has been described that in inflammatory diseases such as atherosclerosis, Gremlin 1 (GREM1), is an inhibitor of monocyte chemotaxis (Muller et al., 2013, 2014). Therefore, its negative regulation could favor chemotactic activity in monocytes of patients with TB, which is critical in vascular inflammation.

### Canonical Pathways and Network Analysis of DEGs

**Figure 4:**
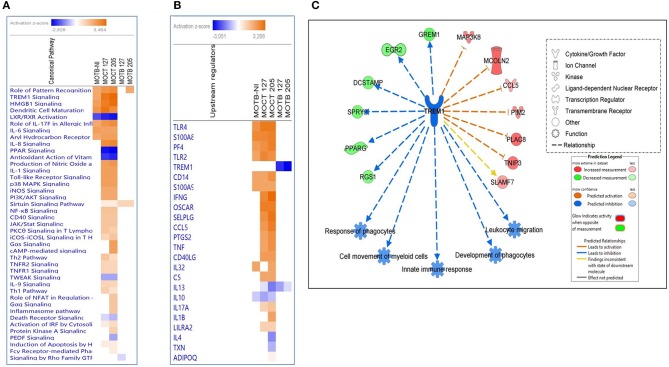
Comparison of significant canonical pathways and upstream regulators in monocytes infected or non-infected with Mtb UT127 and UT205 isolates. Top enriched **(A)** pathways and **(B)** upstream regulators in MoTB and MoCT infected with *M. tuberculosis*. **(C)** Inhibition of TREM1 signaling pathway by MoTB in response to *M. tuberculosis* infection. Pathways are ranked according to the enrichment score (Fisher's exact test *P* ≤ 0.05), and the *Z*-score that predicts activation (≥2) or repression (≤ 2).

An IPA analysis was conducted to identify networks, canonical pathways, and molecular and cellular functions that may differ (*p* < 0.01) between MoCT y MoTB infected with Mtb UT127 and UT205. Supplementary Table 3 shows a list of all the enriched pathways for each condition. A comparative analysis was used to visualize the results across all conditions, including the circulating MoTB transcriptome, which will be described as MoTB-NI to differentiate it from the transcriptomes infected with Mtb UT127 and UT205 (MoTB-127 and MoTB-205). Canonical pathways analysis showed that significantly enriched pathways previously described in MoTB-NI (Figure 1B) were also enriched in MoCT infected with UT127 and UT205. However, MoCT showed higher Z-score values, particularly in response to infection with UT205, confirming the strong response induced with this clinical isolate (Figure 4A). In contrast, few canonical pathways were enriched in MoTB infected with UT127 and UT205. Only the activation of the “Sirtuin 1 signaling pathway” in response to infection with clinical isolates of Mtb is highlighted, which showed higher significant Z-score values in MoTB compared to MoCT (Figure 4A). MoCT-127 and MoCT-205 showed the common activation of pathways involved in bacterial recognition by host cells such as “Toll-like receptor signaling” “Activation of IRF by cytosolic pattern recognition receptors” and “Role of pattern recognition receptors in recognition of bacteria and viruses.” Several common pathways between MoCT-127 and MoCT-205 were inhibited as “LXR/RXR Activation, “PPAR Signaling, “Antioxidant Action of Vitamin C, “TWEAK Signaling,” and “Death Receptor Signaling.” Of note, some canonical pathways were exclusively activated in MoCT-205 such as “Gαs Signaling,” “cAMP-mediated signaling,” “Role of NFAT in Regulation of Immune Response,” “Gαq Signaling” “Inflammasome pathway,” “Protein Kinase A Signaling,” “Fcγ Receptor-mediated Phagocytosis in Macrophages and Monocytes,” and “PEDF Signaling,” which was inhibited (Figure 4A).

Also, the upstream analysis revealed the activation and inhibition of essential molecules, receptors, and transcriptional regulators during TB, being some of these expressed in our database (Figure 4B). Several upstream regulators activated in MoTB-NI were also activated MoCT-127 and MoCT-205 (Figure 4B), while *IFNG, OSCAR, SELPLG, CCL5, PTGS2, TNF*, and *CD40LG* were activated exclusively in MoCT infected with Mtb.

One of the most significantly enriched pathways with a high activation Z-score, except in MoTB infected with Mtb, was the TREM1 (Triggering Receptor Expressed on Myeloid Cells 1) signaling pathway (Figure 4A). TREM1 plays a critical role in the regulation of innate immunity by fine-tuning the inflammatory response and in the modulation of the adaptive immune response (Arts et al., 2013). The upstream analysis showed that TREM1 appears inhibited with the highest negative Z-score in MoTB infected with Mtb, suggesting that immune alterations in TB patients may affect this pathway and thus may play an essential role in the immunopathogenesis of the active disease (Figure 4B). Figure 4C shows the genes up and down-regulated inhibiting of the TREM1 signaling pathway in MoTB in response to Mtb infection.

**Figure 5:**
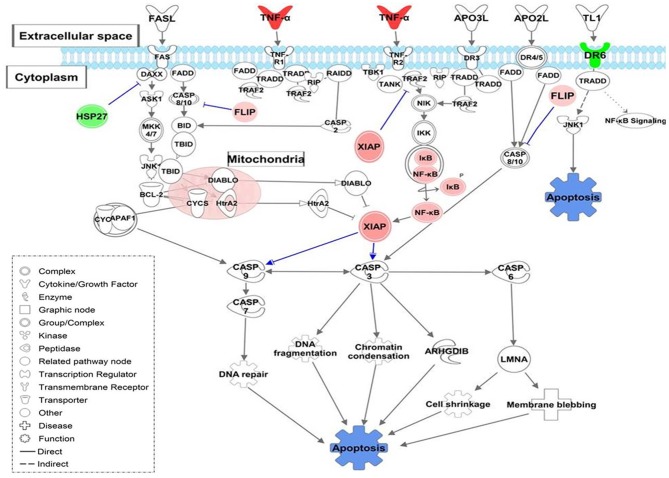
Death Receptor Signaling pathway is illustrating inhibition of apoptosis in monocytes infected with Mtb. The canonical pathway indicates the down-regulated genes (green) and up-regulated genes (Red) in monocytes infected with Mtb. The uncolored nodes are the genes inferred by IPA from their knowledge base. The intensity of color corresponds to an increase in fold change levels. The network was generated with IPA (Fisher test, *P* = 0.05). HSP27 (*HSPB1*), DR6 (*TNFRSF21*), XIAP (*BIRC3*), FLIP (*CFLAR*), NF-κB (*NFKB1*), IKB (*NFKBIA*).

Also, MoTB and MoCT in response to both UT127 and UT205 showed inhibition (negative Z-score) of the canonical pathway “Death Receptor Signaling Pathway” (Figure 5). The inhibition of “Death Receptor Signaling Pathway” was associated with up-regulation of genes such as *CFLAR* (*FLIP*), *BIRC3* (*XIAP*), *NFKB1, NFKBIA*, which have been previously described as anti-apoptotic genes (Ilbäck et al., 2004), and with the down-regulation of the *TNFRSF21* gene (*DR6*, death receptor-6), which is directly associated with the inhibition of cell death by apoptosis (Figure 5).

**Figure 6:**
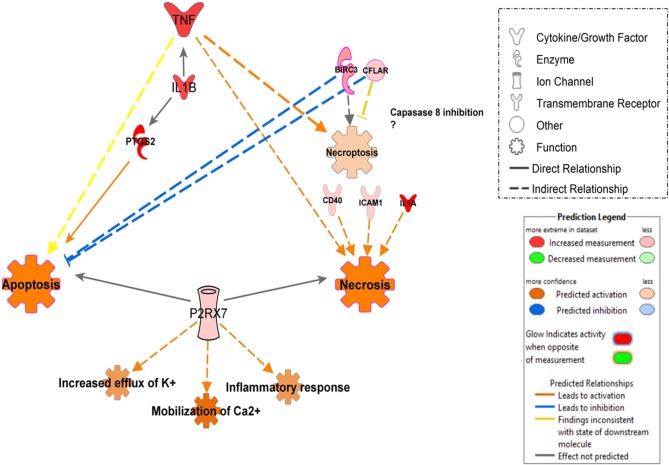
Proposed model of signal pathways that modulate cell death in monocytes infected with Mtb UT127 and UT205. Mycobacterial recognition by infected monocytes can trigger different programs of cell death may be induced by the secretion of pro-inflammatory mediators in response to infection. *TNF* induces apoptosis, necrosis, and necroptosis. The induction of necroptosis via *TNF* could occur when *CFLAR* and *BIRC3* genes inhibit caspase-8. *IL1B* enhances the production of *TNF* and *PTGS2* that induces apoptosis. Additionally, the rapid intracellular growth of virulent mycobacteria results in massive monocyte damage. The extracellular ATP (eATP) released by necrotic cells engage the *P2X7R* on their surfaces on neighboring cells. P2RX7 signaling cooperates with mycobacterial components exhibiting the membrane-lysing activity and accelerates the necrotic death of infected monocytes. On the other hand, low amounts of eATP induce apoptotic cell death by a mechanism dependent on P2RX7 activation. Thus, apoptosis induction of infected monocytes favors mycobacterial killing, whereas necrotic cell death facilitates the spread of the bacilli. We suggest that monocyte infection with UT127 is associated with apoptosis, while infection with UT205, although it can induce death by apoptosis, the exclusive expression of the *P2RX7* and *CFLAR* genes suggests induction of death by necrosis or necroptosis. Up-regulated (red) and down-regulates (green) genes are highlighted and the color depth is correlated to the fold change. Inhibition (displayed in blue) or activation (displayed in orange) of functions are based on IPA activation z-score ≥ 2 and the network was generated with IPA (Fisher test, *P* ≤ 0.05).

In contrast, in additional analysis, we showed that infection with UT127 could induce the expression of genes such as *TNF, IL1B*, and *PTGS2*, which together induce activation of apoptosis in MoCT but not in MoTB. Interestingly, infection with the clinical isolate UT205 exclusively induced the expression of the *P2RX7* gene, which with other genes such as *CFLAR* and *BIRC3*, showed enrichment in processes of cell death by necrosis and necroptosis (Figure 6).

### Technical Validation of Differential Expression by Quantitative Real-Time PCR

We selected 9 genes that were up-regulated in MoTB-NI by microarray analysis (*IL1B, CXCL10, PTGS2, TNF, TNFAIP6, SERPINB2, CCL20, IL6*, and *IL8*), and their expression levels were assessed by quantitative PCR analysis using the same samples utilized in the microarray experiment (Supplementary Figure 4A). The relative expression results by qRT-PCR of the 9 genes showed that these are significantly up-regulated (Supplementary Figure 4A). The relative expression levels of each gene were transformed to Log_2_ and compared with the expression values obtained by microarray, showing an explicit agreement between the two techniques (Supplementary Figure 4B), confirming our results with the microarray analysis.

## Discussion

Monocytes play a significant role during infection and inflammation. They are recruited to the infection foci, where they participate in infection control and promote a protective inflammatory response leading to re-establish homeostasis. Also, monocytes can differentiate to inflammatory macrophages and dendritic cells, expanding and potentiating a protective immune response (Shi and Pamer, 2011).

Nevertheless, inflammatory monocytes can also play a deleterious effect by fueling and perpetuating the inflammatory response leading to unrestricted control of microbial infection and tissue pathology (Shi and Pamer, 2011; Pahari et al., 2018; Sampath et al., 2018). Our group and others have reported phenotypic and functional alterations in monocytes from TB patients compared to healthy controls (Sanchez et al., 2006; Balboa et al., 2011; Castaño et al., 2011a,b; Lastrucci et al., 2015). However, global studies on monocytes from TB patients and the consequences on Mtb infection aimed to understand better the innate immune response to Mtb infection are scarce (Esterhuyse et al., 2015; Naranbhai et al., 2015). So far, no study on the mRNA expression profile of monocytes from TB patients to Mtb infection has been reported. In this study, using the microarray technology, we first addressed the transcriptomic differences of circulating MoTB compared to MoCT, and second, we analyzed the consequences on the mRNA profiles of MoTB and MoCT to infection with two clinical isolates of the Latin American and Mediterranean (LAM) family of Mtb.

### Circulating Monocytes From TB Patients Show a Transcriptional Profile Associated With Inflammation

Cytokines and chemokines are essential players in the immune response to mycobacterial infections (Jo et al., 2003). Pro-inflammatory cytokines, including IL-1β, IL-1α, IL-6, IL-8, and TNF-α, are responsible for both the protection and pathogenesis of the human host with active TB. Elevated levels of TNF-α, IL-1β, and IL-6 may be responsible for exaggerated inflammation and the deleterious effects associated with TB (Flynn et al., 2011; Joshi et al., 2015). Spontaneous production of these pro-inflammatory cytokines by monocytes was observed in active TB (Toossi et al., 1995), and associated with activation of monocytes *in vivo* due to circulating mycobacterial products, which may be dependent on the severity status of the disease (Barnes et al., 1992; Dlugovitzky et al., 1995; Sethna et al., 1998; Banerjee et al., 2003). For example, the 30-kDa antigen of Mtb activates monocytes from TB patients to produce CCL20, which is modulated by TNF via a MAPK/NF-κB-mediated transcriptional mechanisms (Lee et al., 2008). CCL20 is considered an essential link between innate and acquired immunity. In agreement with these results, we also found high expression values of *CCL20* in MoTB compared with MoCT. Given that CCL20 participates in the first steps of adaptive response, this result may suggest a critical role of *CCL20* in the immunopathogenesis of TB.

On the other hand, we observed that MoTB gene signature was significantly enriched in the canonical pathway “Hematopoiesis from pluripotent stem cell” (Figure 1D), and biological functions such as “Development of hematopoietic progenitor cells,” “Monocytopoiesis,” “Development of mononuclear leukocytes” and “Migration of bone marrow cells,” which were associated with up-regulated genes (*TNF, IL6*, and *IL7R*) and the *CD74* gene, which was down-regulated (Supplementary Table 3).

During severe systemic infection with intracellular pathogens (e.g., Mtb), hematopoiesis is changed to demand-adapted myelopoiesis, resulting in increased myeloid progenitor proliferation and therefore an enhanced production of monocytes, that is, “emergency monocytopoiesis” (Boettcher and Manz, 2016). During active TB, monocytosis depends on the increased monocytopoiesis and the premature release of monocytes from the bone marrow, leading to accumulation of recruited immature monocytes at the sites of infection, contributing to the pathogenesis of the disease (Schmitt et al., 1977; Fenton and Vermeulen, 1996; Ingersoll et al., 2011). An increased alveolar macrophage death may cause this monocytopoiesis due to the high toxicity of tubercle bacilli (Schmitt et al., 1977).

In addition to being activated by pathogen products, hematopoietic stem cells (HSCs) also respond to inflammatory cytokines produced in response to infection by pathogens and to other signaling pathways in the bone marrow microenvironment (Baldridge et al., 2011). It is unclear whether these factors are produced in the lungs of TB patients at the levels sufficient to have systemic effects. Interestingly, mycobacteria can be isolated from the bone marrow in different circumstances, and recent reports highlighted that Mtb resides latently in the bone marrow and stem progenitor cells (Das et al., 2013; Reece et al., 2018). However, the mechanisms influencing monocyte development and maturation during TB remains mostly undefined.

Another alteration observed in monocytes of our patients with TB was the negative regulation of genes encoding MHC II molecules such as *CD74, HLA-DPA1, HLA-DRB3*, and *HLA-DRB4*, which were grouped in the functional category “Antigen processing and presentation” (Figure 1A). The reduced expression of HLA-DR on monocytes has been associated with the severity of infectious diseases since HLA-DR directly influences cell activation and increases the efficiency of antigen presentation to T lymphocytes (Lapa e Silva et al., 1996; Ditschkowski et al., 1999). Our group had previously reported that patients with TB have a higher proportion of inflammatory circulating monocytes (Castaño et al., 2011b), and they express lower amounts of surface HLA-DR (Sanchez et al., 2006). Also, surface HLA-DR expression was restored to normal levels after anti-TB treatment (Sanchez et al., 2006), suggesting that the systemic effects of the active disease could be explained at least in part by differences the monocyte phenotype and function between TB patients and healthy controls. Strengthening this hypothesis, data from the present study suggest that monocytes from TB patients negatively regulated the expression of genes encoding MHC II molecules, possibly as a systemic consequence of the active TB. Bacterial or host-derived factors may be responsible for such a response. Overall, these data suggest that circulating monocytes from TB patients both respond and contribute to maintaining a pro-inflammatory milieu in TB patients.

### MoTB *in vitro* Infected With Mtb Show an Attenuated Pro-Inflammatory Profile Compared With MoCT

Our results indicate that circulating monocytes from TB patients display an altered transcriptional profile. This alteration may due to the inflammatory milieu associated with TB disease, which alters the expression pattern of monocytes. However, when infected with UT127 and UT205, the pro-inflammatory expression profile observed in circulating monocytes from MoTB was absent. This attenuated pro-inflammatory response could be due to a negative feedback mechanism to control the exacerbated inflammatory process occurring in TB patients triggered by the first contacts of the mononuclear phagocytes with mycobacteria or its products (Pereira et al., 2004).

A relevant pathway that was highly induced in circulating MoTB was the TREM1 signaling pathway (Figure 1B). Interestingly, this pathway was inhibited once MoTB were infected *in vitro* with both clinical isolates of Mtb (Figure 4). TREM1 is expressed on neutrophils, monocytes, and tissue macrophages and is implicated in the propagation/amplification of inflammation induced by stimulation of TLRs in the pathogenesis of many infectious diseases (Bouchon et al., 2000; Colonna and Facchetti, 2003). In primary human monocytes, TREM1 activation did not trigger innate antimicrobial pathways directed against intracellular Mtb but led a robust production of pro-inflammatory cytokines such as IL8 and TNF-α (Bleharski et al., 2003), which could be consistent with the inflammatory response found in circulating MoTB. In concordance, we found that in circulating MoTB, *TREM1* signaling also showed a significant overlap with TLRs (TLR2 and TLR4) and with effector cytokines, whose expression is induced by TREM1 activation (Figure 1B). Moreover, the activation of TREM1 in association with TLRs signaling led to the significant enrichment of functions associated with the activation of myeloid cells, as previously found in an integrative study of eight microarray datasets from the blood of active TB patients (Joosten et al., 2013).

Furthermore, the pronounced myeloid signature resulted from increased numbers of inflammatory monocytes and increased gene expression in purified monocytes (Berry et al., 2010). The activation of TREM1 and TLRs signaling associated with myeloid functions may also be a result of *in vivo* priming by circulating Mtb antigens. The activation of the TREM1 signaling pathway by MoCT infected with Mtb could confirm our hypothesis that Mtb antigens modulate this pathway. It has been speculated that pathogen-associated molecular patterns (PAMPs) could directly activate TREM1 (Arts et al., 2013). Mtb could also be recognized by TREM1 and amplify the inflammatory response initiated by TLRs engagement.

Remarkably, the TREM1 signaling pathway was inhibited when circulating MoTB were infected *in vitro* with clinical isolates of Mtb. We speculate that since monocytes could be previously activated by circulating Mtb antigens, the innate immune response could be over-activated (Bhattacharya and Matthay, 2013). Since pathogen recognition signals need to be controlled to prevent tissue damage, the inhibition of TREM1 signaling found in MoTB in response to infection with both clinical isolates of Mtb may be a mechanism to control the consequences of chronic inflammation associated to active TB disease. It has been shown that the blockade of TREM1 reduces inflammation and increases survival in animal models of bacterial infections that cause systemic inflammatory syndromes (Colonna and Facchetti, 2003). However, the inhibition of the TREM1 signaling pathway in MoTB infected with Mtb could reflect an impaired recognition of the pathogen due to its previous activation in circulation, and therefore fail to activate adequate defense mechanisms that control the infection. This inhibition could correlate with the attenuated innate immune response observed in MoTB infected with Mtb, as well as the down-modulation of genes that were associated with the inhibition of the differentiation of monocytes and the negative regulation of endocytosis and phagosome maturation (Figures 3C,D).

### Transcriptomic Response to UT127 and UT205 Suggests Differences in Virulence

Monocyte responses to infection can also be modulated by differences in virulence, the circulating Mtb strains, and the genetic characteristics of the infected population. However, the response of monocytes to circulating virulent clinical isolates of Mtb is just beginning to be examined. In general, reported findings to suggest that circulating strains of Mtb induce varied immune responses (differences in cytokine production, intracellular replication, type and extent of monocyte/macrophage cell death) compared to H37Rv considered as a laboratory strain of Mtb (Carmona et al., 2013; Tientcheu et al., 2017).

A previous report from our laboratory, based on phenotypic differences of human monocytes and macrophages in response to infection with two Colombian LAM09 (UT127 and UT205) clinical isolates, suggested differences in virulence between these closely related strains (Duque et al., 2014). Several strains of Mtb have been identified that show increased virulence in animal models compared to reference strains such as H37Rv, and prominent among these are members of the W-Beijing family (Carmona et al., 2013). Our group sequenced the complete genome of the Colombian clinical isolate UT205 (Isaza et al., 2012), the first complete genome sequenced, assembled, and annotated in Colombia. This isolate was collected as part of the activities of the CCITB in 2005–2009 during the study and follow-up of a cohort of infected individuals living with patients with pulmonary TB (del Corral et al., 2009). In comparison with Mtb H37Rv as a reference genome, a 3.6 kb genomic deletion was found that affects several genes belonging to the *dosR* region. The genes encoded by this region participate in the adaptation of Mtb to different situations of stress, allowing the bacteria to enter the state of quiescence (“dormancy”), which is supposed to occur during latent TB (Boon and Dick, 2012). In contrast, the isolate UT127 does not have this deletion (Baena et al., 2019).

Of interest, a comparison of the *in vitro* response to infection with the Colombian clinical isolates UT205 and UT127, using human monocytes and different populations of human macrophages, showed a different capacity for the induction of cell death, particularly by necrosis, and the production of cytokines (Duque et al., 2014). The UT205 isolate induced a more considerable amount of necrosis and fewer cytokines, among others of TNFα, compared to the UT127 isolate, suggesting differences in virulence that could partially explain its epidemiology (Duque et al., 2014). While the UT205 isolate was transmitted in the household contact and presumably resulted in an incident case, the UT127 isolate was not transmitted to the household contact, in which no infection was detected using a homemade IGRA (del Corral et al., 2009).

More recently and based on previous results, our group (Baena et al., 2019) compared the transcriptional response of both clinical isolates (UT127 and UT205) under two axenic media conditions. The results of this study showed that the clinical isolate UT205 focus mainly in the activation of virulence systems such as the ESX-1, synthesis of diacyl-trehalose, polyacyltrehalose, and sulfolipids, while UT127 concentrates its efforts mainly in the survival mode by the activation of the DNA replication, cell division, and lipid biosynthesis.

We found some differences in the transcriptomic responses induced by UT127 and UT205 in MoTB or MoCT, which could be due to differences in virulence. For example, in response to infection with UT127, MoTB exclusively expressed 31 genes (7 up-regulated and 24 down-regulated) (Figure 2A). These genes were mainly associated with the negative regulation of processes associated with endocytosis and phagosome maturation, such as protein localization to endosome, actin cytoskeleton organization, and cell projection assembly (Figure 3D). A main primary function of professional phagocytes is the uptake of microorganisms through phagocytosis. Therefore, our data suggest that Mtb UT127 infecting MoTB could use this mechanism to create a more favorable intracellular environment for survival by inhibition of phagosome-endosome interactions.

Other differences found between UT127 and UT205 were the induction of the expression of genes associated with the category of Cell death. An essential mechanism of Mtb pathogenesis is the ability to control cell death pathways in infected cells and to down-regulate immune responses of host cells using various strategies (Halder et al., 2015). For example, virulent mycobacteria can negatively modulate the apoptotic cell death through the down-regulation of pro-apoptotic genes or the up-regulation of anti-apoptotic genes in the host (Oddo et al., 1998; Rodrigues et al., 2013). Although both strains showed modulation of the inhibition of cell death by apoptosis through the expression of anti-apoptotic genes, and the negative regulation of the pro-apoptotic gene *TNFRSF21/DR6* (Figure 5), an additional analysis showed that infection with UT127 could also induce the expression of genes such as *TNF, IL1B*, and *PTGS2*, which could be associated with other cell death mechanisms other than apoptosis, such as necroptosis; while infection with the clinical isolate UT205 exclusively induced the expression of the *P2RX7* gene, which along with other genes such as *CFLAR* and *BIRC3*, showed enrichment in processes of cell death mainly by necrosis (Figure 6).

Although the molecular mechanism of DR6 action, including its immunological ligand(s), is unknown in TB, we suggest that Mtb could modulate its down-regulation as an attempt to protect itself from monocyte apoptosis and promote its dissemination. On the other hand, the up-regulation of the *P2X7* gene (P2X7 purinergic receptor), which was associated with the activation of functions such as increased efflux of K^+^, mobilization of Ca2^+^ and inflammatory response (Figure 6). Among the purinergic receptors, P2X7R recognizes eATP and is uniquely implicated in the induction of pro-inflammatory cytokine production and cell death (Miller et al., 2011). ATP is released by necrotic cells as a result of plasma membrane permeabilization as well as by apoptotic cells through pannexin-1 hemichannels (Junger, 2011). P2X7R engagement allows the influence of Ca2^+^ and efflux of K^+^, culminating in the cytosol ionic disturbance that triggers the NLRP3-inflammasome and promotes the release of active IL-1β (Di Virgilio et al., 1998). The magnitude and duration of the stimulus determine whether P2X7R activation induces apoptotic or necrotic cell death (Mariathasan et al., 2006). Higher amounts of eATP associated with P2X7R signaling may induce necrotic death of macrophages infected with Mtb while lower amounts may induce apoptosis (Amaral et al., 2016). Based on these results, we constructed a model that could explain how two strains of Mtb (UT127 and UT205) with differential virulence could modulate different cell death mechanisms in monocytes (Figure 6).

Altogether, our data revealed enrichment for genes involved in apoptosis, regarded as a host innate immune mechanism contributing to limiting and containing mycobacterial growth following infection (Keane et al., 1997; Behar et al., 2011). The induction of anti-apoptotic genes may, however, represent a strategy employed by the pathogen to limit apoptosis, enabling survival and replication within the monocyte. In our experimental system, monocytes were infected with a high MOI (10:1), combined with the fact the infection time was relatively short (6 h). Since the cell death type may vary depending on factors as the “burst size” of intracellular bacterium loads, bacterial preparation (opsonized or not), time-course of infection, bacterial virulence and host cell type (Amaral et al., 2016), it might be challenging to know the precise a clear association of the genes found here on the induction of a particular type of cell death.

Also, the infection with UT205 of MoCT showed a strong response through a signature of 86 genes, which was absent in MoTB. This gene expression signature was associated with IFN-inducible genes such as type I (IFNα/β) and type II (IFN-γ) interferon signaling pathway (*IRF1, IFIT2, ISG20, GBP1, GBP2, GBP5, MX1, MT2A*) (Figure 3B, Supplementary Table 4). The mechanisms that mediate disease exacerbation mediated by type I IFN are not yet fully known and presumed to be complex. The increase in migration of inflammatory monocytes and neutrophils to the lungs of mice infected with Mtb has been suggested to be dependent on type I IFN (Dorhoi et al., 2014). On the other hand, transcriptomic analyses of peripheral blood mononuclear cells from TB patients revealed a dominant IFN gene signature, including those responding to type I and typed II (IFN-γ) signaling (Berry et al., 2010; Maertzdorf et al., 2012; Ottenhoff et al., 2012; Cliff et al., 2013). This gene expression signature correlated with the radiographic extent of the disease and diminished upon anti-tuberculous treatment (Maertzdorf et al., 2012; Bloom et al., 2013; Cliff et al., 2013). Taken together, these findings suggest that the virulence of UT205 may contribute to disease progression through the induction of the signature of IFN-inducible genes as a mechanism to subvert host immunity and establish disease.

The low amount of samples used in this pilot study is a primary limitation precluding us to define whether our observations extend to many other cases of people with active TB from different ancestry and/or infected with clinical strains of the LAM family or other families of Mtb, and current findings need to be replicated in future studies using a larger number of samples. However, the sample size used was sufficient to achieve statistical significance between the studied groups based on the stringent protocol used to select the differentially expressed genes. Although microarrays present quantitative and qualitative limitations compared with more robust methods such as RNA-seq, its analytical platform is very well-developed to generate confident results.

In conclusion, the high levels of gene expression of pro-inflammatory cytokines suggest that patients were in an intense inflammatory state with pre-activation of monocytes *in vivo* when compared with healthy controls. These results allow us to conclude that the signature gene expression in peripheral blood could serve as an indicator of risk of TB and in part, derive from enhanced expression of pro-inflammatory transcripts in the monocyte population, suggesting that systemic monocyte activation, in addition to a possible increased monocyte abundance (monocytosis), may comprise a hallmark of TB progression during active disease.

Our data show that the transcriptional pattern observed in monocytes infected with clinical isolate depended on the cellular origin (MoCT or MoTB) as well as on the differences in virulence between both clinical isolates of Mtb (UT127 or UT205). Thus, in response to infection with Mtb UT127 and UT205, MoTB were unable to express genes associated with infection control and showed an attenuated gene expression compared to the response to infection observed in MoCT. The attenuation of the immune response observed in MoTB infected with Mtb reconfirms our hypothesis that the pre-activation of monocytes (systemic effect of TB) alters their expression pattern after the first contact with Mtb or its products. Therefore, its transcriptional response is constituted as a mechanism of the host to decrease and control the excess inflammatory response during active disease. Thus, we speculated that when monocytes are recruited to the lung during active TB, their differentiation and acquisition of myeloid effector functions could be dramatically altered, suggesting that they could be ineffective in helping the macrophage-mediated killing of intracellular mycobacteria. Therefore, we conclude that these monocytes could contribute to the pathogenesis of TB. However, the TB blood transcriptional signature could represent altered cell composition or changes in discrete cellular populations (as monocytes), which could bias their contribution to the gene expression profile.

## Data Availability Statement

The datasets generated for this study can be found in the GEO (Gene Expression Omnibus), accession GSE139871.

## Ethics Statement

This study was approved by the Ethical Committee of the Faculty of Medicine, Universidad de Antioquia, Colombia, and by the Ethical Committee of Clínica Cardiovascular Santa María, Medellín, Colombia. Written consent was voluntarily signed by all pulmonary TB patients (PTB) and control subjects (CT) before sample acquisition. The patients/participants provided their written informed consent to participate in this study.

## Author Contributions

LL performed the experiments, collected and analyzed the data, interpreted the data, and wrote the manuscript. HO collected and analyzed the data. LB designed the study, interpreted the data, and wrote the manuscript.

## Conflict of Interest

The authors declare that the research was conducted in the absence of any commercial or financial relationships that could be construed as a potential conflict of interest.
