# Supplementary material for: Infection of Monocytes From Tuberculosis Patients With Two Virulent Clinical Isolates of Mycobacterium tuberculosis Induces Alterations in Myeloid Effector Functions
Source: Front Cell Infect Microbiol. 2020 Apr 23;10:163. doi: 10.3389/fcimb.2020.00163 (PMC7190864; doi:10.3389/fcimb.2020.00163)
Supplement: Supplementary file 1 [file Data_Sheet_1.pdf]

Supplementary Figures

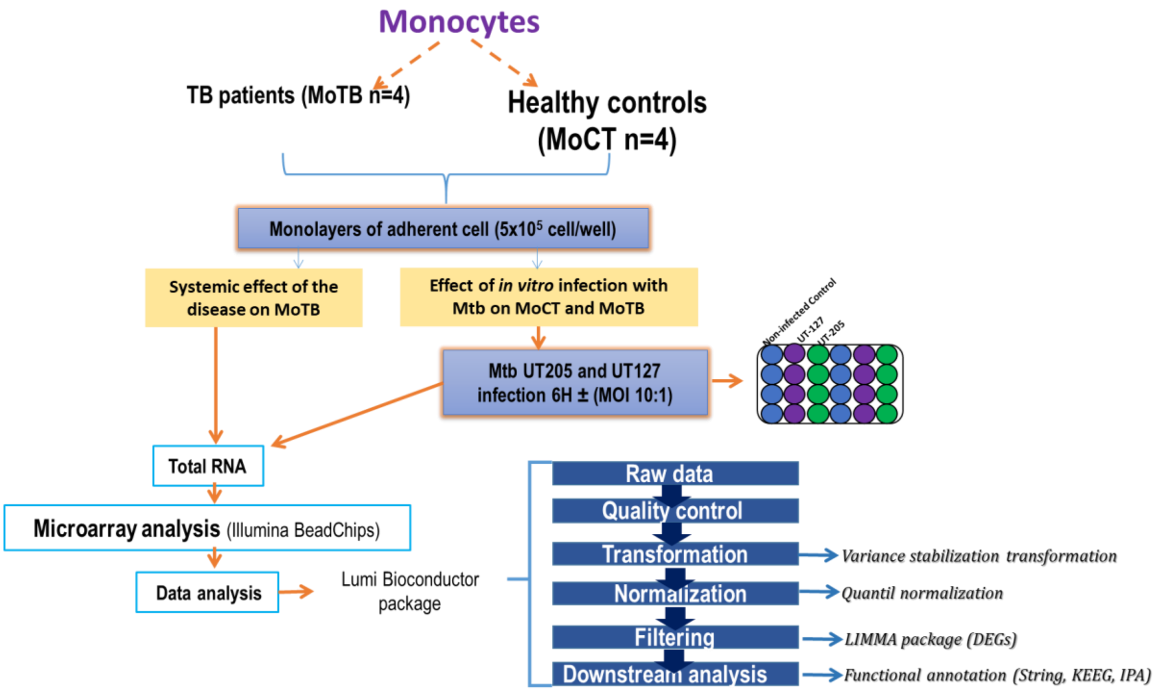

Supplementary Figure 1. ***Experimental outline***. Monocytes from healthy control subjects (MoCT) and pulmonary TB patients (MoTB) were investigated. For the systemic effects of the disease, total RNA from circulating monocytes was extracted. To investigate the effects of M. tuberculosis infection, monocyte monolayers were infected with a MOI of 10:1 for 6 hours, and total RNA was extracted. Then, labeled cRNA samples were hybridized to Illumina Human BeadChips. The raw data was then processed by the Lumi Bioconductor package using the R software. After normalization, the differentially expressed genes (DEGs) were obtained by the Limma package, and functional annotation performed with String, KEGG and IPA (for further details see Materials and Methods).

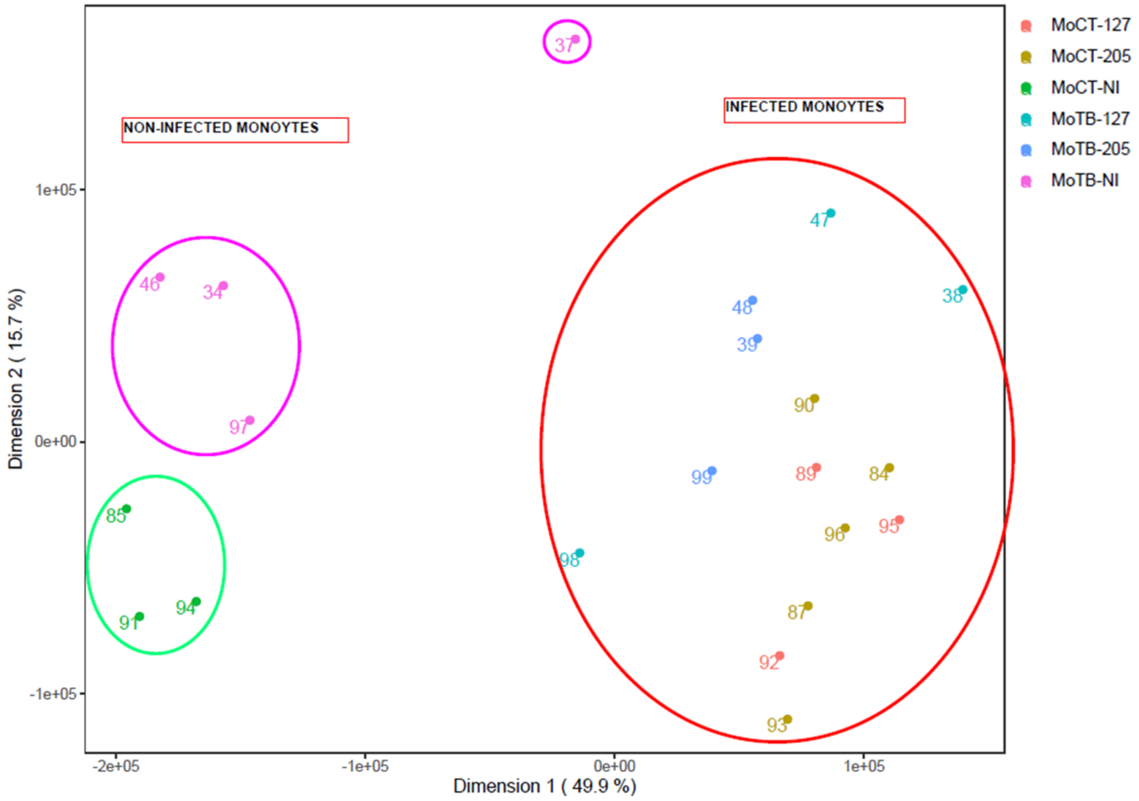

Supplementary Figure 2. Multidimensional scaling plot of non-infected (MoCT-NI, MoTB-NI), and *M. tuberculosis* (Mtb)-infected monocytes from healthy subjects (n=4) (MoCT-127, MoCT-205) and pulmonary tuberculosis (TB) patients (n=4) (MoTB-127, MoTB-205), based on microarray data.

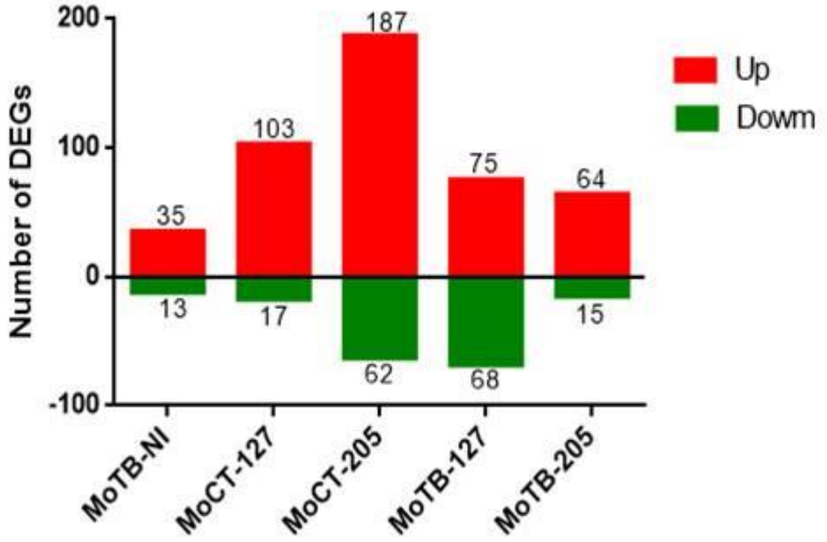

Supplementary Figure 3. Number of DEGs in MoTB-NI versus MoCT, and DEGs from MoTB and MoCT infected with clinical isolates of Mtb (UT127 and UT205). DEGs were obtained relative to their uninfected controls: 1) (MoTB-NI vs MoCT-NI), 48 DEGs; 2) (MoCT-NI vs MoCT-127), 120 DEGs; 3) (MoCT-NI vs MoCT-205), 249 DEGs; 4) (MoTB-NI vs MoTB-127), 143 DEGs; 5) (MoTB-NI vs MoTB-205), 79 DEGs. Red color indicates up-regulation and green color indicates down-regulation (1.5 log2-fold, p≤0.05).

(A)

(B)

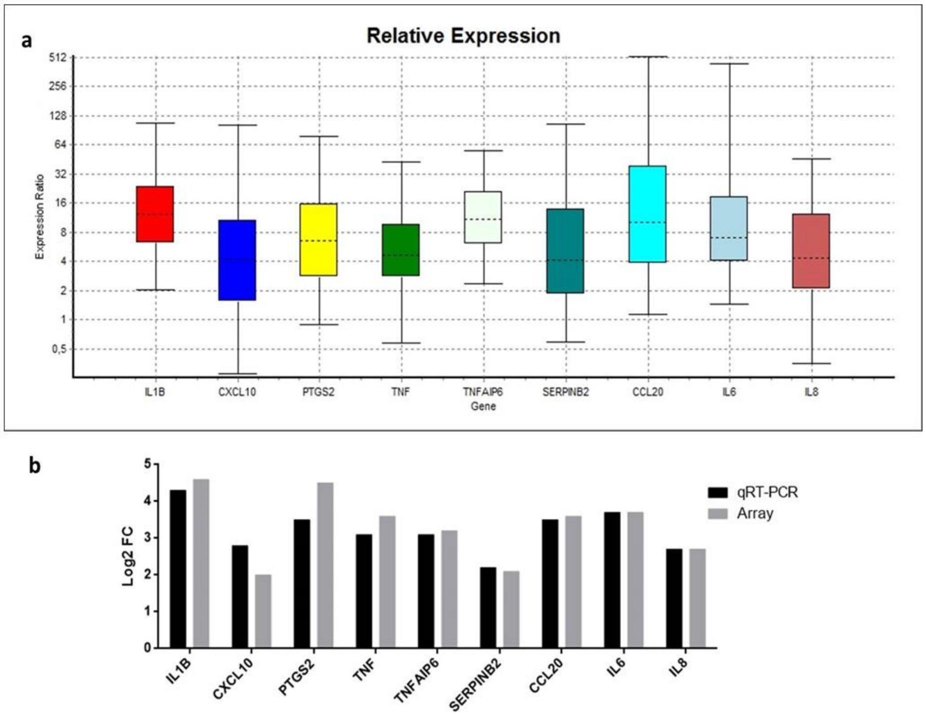

Supplementary Figure 4: ***Validation of differential expression by quantitative real-time PCR***. (**A**) Relative expression of *IL1B*, *CXCL10*, *PTGS2*, *TNF*, *TNFAIP6*, *SERPINB2*, *CCL20*, *IL6* and *IL8* from MoTB compared to MoCT by qRT-PCR. The genes of interest were normalized by using the housekeeping gene β-actin. The expression values indicate the fold induction for a duplicate for three different donors. (**B**) Comparison between expression data for both Illumina array and qRT-PCR of the genes of interest.
