# Supplementary material for: Infection of Monocytes From Tuberculosis Patients With Two Virulent Clinical Isolates of Mycobacterium tuberculosis Induces Alterations in Myeloid Effector Functions
Source: Front Cell Infect Microbiol. 2020 Apr 23;10:163. doi: 10.3389/fcimb.2020.00163 (PMC7190864; doi:10.3389/fcimb.2020.00163)
Supplement: Supplementary file 2 [file Data_Sheet_2.zip › Table S1.pdf]

Supplementary table 1: Sequences of primers used for the validation of differential expression by quantitative real-time PCR.

Gene	Primer (5'--3')		Length	Author
TNFAIP6	Forward	TCCATATGGCTTGAACGAGCAG	84	Coldren *et al*., 2003
	reverse	CTTAGCTTCTGCGTAGGTGAGC		
TNF	Forward	AGGCGGTGCTTGTTCCTC	167	Volpe *et al*., 2006
	reverse	GTTCGAGAAGATGATCTGACTGCC		
ACTB	Forward	CATCGAGCACGGCATCGTCA	211	Ishii *et al*., 2006
	reverse	TAGCACAGCCTGGATAGCAAC		
SERPINB2	Forward	CACCCAGAACCTCTTCCTCTCC	134	Bakshi *et al*.,2008
	reverse	TAACTGCATTGGCTCCCACTTC		
PTGS2	Forward	CGGTCCTGGCGCTCAG	110	Tomlinson *et al*., 2012
	reverse	CCGGGTACAATCGCACTTATACTG		
CXCL10	Forward	GTGGCATTCAAGGAGTACCTC	198	Fu *et al*., 2013
	reverse	TGATGGCCTTCGATTCTGGATT		
IL8	Forward	ACTGAGAGTGATTGAGAGTGGAC	112	Fu *et al*., 2013
	reverse	AACCCTCTGCACCCAGTTTTC		
IL1B	Forward	TTCTTCGACACATGGGATAACG	169	Zhang *et al.*, 2014
	reverse	TGGAGAACACCACTTGTTGCT		
CCL20	Forward	AAGTTGTCTGTGTGCGCAAATCC	107	Reyes *et al*., 2015
	reverse	CCATTCCAGAAAAGCCACAGTTTT		
IL6	Forward	AGACAGCCACTCACCTCTTCAG	132	Reyes *et al*., 2015
	reverse	TTCTGCCAGTGCCTCTTTGCTG		
