# Supplementary material for: Infection of Monocytes From Tuberculosis Patients With Two Virulent Clinical Isolates of Mycobacterium tuberculosis Induces Alterations in Myeloid Effector Functions
Source: Front Cell Infect Microbiol. 2020 Apr 23;10:163. doi: 10.3389/fcimb.2020.00163 (PMC7190864; doi:10.3389/fcimb.2020.00163)
Supplement: Supplementary file 2 [file Data_Sheet_2.zip › Table S2.pdf]

Supplementary table 2: Full list of DEGs for MoTB *in vitro* non-infected (NI) and MoTB and MoCT *in vitro* infected with Mtb UT127 and UT205.

List of DEGs in MoTB-NI compared to MoCT-NI

Gene	Gene Name	LogFC	P.Value	FDR
AMFR	autocrine motility factor receptor. E3 ubiquitin protein ligase	1.8	1.6E-03	0.96
ATP9A	ATPase. class II. type 9A	1.6	5.5E-04	0.42
CCL1	chemokine (C-C motif) ligand 1	2.7	9.4E-03	0.75
CCL20	chemokine (C-C motif) ligand 20	3.6	5.4E-04	0.42
CCL24	chemokine (C-C motif) ligand 24	-1.5	1.7E-02	0.98
CCL3L1	chemokine (C-C motif) ligand 3-like 1	1.6	1.5E-02	0.98
CCL4L1	chemokine (C-C motif) ligand 4-like 1	2.1	3.6E-02	0.89
CCL5	chemokine (C-C motif) ligand 5	1.8	1.1E-02	0.77
CCL8	chemokine (C-C motif) ligand 8	2.8	2.5E-06	0.03
CD74	CD74 molecule. major histocompatibility complex. class II invariant chain	-1.5	8.0E-03	0.72
CXCL1	chemokine (C-X-C motif) ligand 1	3.3	3.7E-04	0.42
CXCL10	chemokine (C-X-C motif) ligand 10	2.1	5.2E-04	0.42
CXCL2	chemokine (C-X-C motif) ligand 2	2.2	2.4E-02	0.84
CXCL8	chemokine (C-X-C motif) ligand 8	3.1	2.1E-03	0.64
EBI3	Epstein-Barr virus induced 3	1.7	2.4E-02	0.84
FGL2	fibrinogen-like 2	-2.4	1.3E-04	0.33
GJB2	gap junction protein. beta 2. 26kDa	2.1	3.3E-03	0.64
GRAMD1A	GRAM domain containing 1A	1.6	2.4E-02	0.84
GSTM1	glutathione S-transferase mu 1	1.5	9.2E-03	0.75
GSTM2	glutathione S-transferase mu 2 (muscle)	1.8	4.8E-03	0.64
HLA-DPA1	major histocompatibility complex. class II. DP alpha 1	-2.1	6.7E-04	0.42
HLA-DRB3	major histocompatibility complex. class II. DR beta 3	-2.3	6.8E-04	0.42
HLA-DRB4	major histocompatibility complex. class II. DR beta 4	-2.4	5.4E-04	0.42
HLA-DRB6	major histocompatibility complex. class II. DR beta 6 (pseudogene)	-1.6	1.0E-03	0.53
IDO1	indoleamine 2.3-dioxygenase 1	1.9	2.9E-03	0.64
IL1A	interleukin 1. alpha	3.4	3.1E-03	0.64
IL1B	interleukin 1. beta	4.7	7.0E-04	0.42
IL1R2	interleukin 1 receptor. type II	-1.8	3.9E-02	0.89
IL23A	interleukin 23. alpha subunit p19	2.3	1.4E-02	0.80
IL6	interleukin 6	3.8	7.7E-03	0.71
IL7R	interleukin 7 receptor	2.0	1.6E-03	0.62
MARCKS	myristoylated alanine-rich protein kinase C substrate	1.8	1.2E-02	0.77
MT1E	metallothionein 1E	1.6	2.8E-02	0.87
MT1G	metallothionein 1G	1.9	7.1E-03	0.94
MT2A	metallothionein 2A	1.6	4.5E-02	0.90
MX1	MX dynamin-like GTPase 1	1.8	9.3E-03	0.75
PRCP	prolylcarboxypeptidase (angiotensinase C)	-1.5	4.4E-06	0.03
PRKAR1A	protein kinase. cAMP-dependent. regulatory. type I. alpha	-1.7	7.7E-02	0.95
PTGS2	prostaglandin-endoperoxide synthase 2 (cyclooxygenase)	4.5	3.9E-05	0.14
SERPINB2	serpin peptidase inhibitor. clade B (ovalbumin). member 2	2.5	8.1E-03	0.95

Gene	Gene Name	LogFC	P.Value	FDR
SLC25A24	solute carrier family 25 (mitochondrial carrier; phosphate carrier). member 24	1.9	3.7E-03	0.64
SNORA12	small nucleolar RNA. H/ACA box 12	-1.8	5.8E-02	0.93
STAT4	signal transducer and activator of transcription 4	1.6	2.5E-03	0.64
TACSTD2	tumor-associated calcium signal transducer 2	1.7	9.7E-02	0.96
TNF	tumor necrosis factor	3.6	3.7E-03	0.64
TNFAIP6	tumor necrosis factor. alpha-induced protein 6	3.2	4.6E-04	0.42
USF1	upstream transcription factor 1	1.8	1.0E-02	0.76
VAV3	vav 3 guanine nucleotide exchange factor	-2.0	7.4E-03	0.70

List of DEGs in MoCT infected with Mtb UT127 compared to MoCT-NI

Gene	Gene Name	LogFC	P.Value	FDR
ACP5	acid phosphatase 5. tartrate resistant	-2.0	2.1E-05	9.1E-04
ACSL1	acyl-CoA synthetase long-chain family member 1	2.2	1.5E-04	3.2E-03
ADA	adenosine deaminase	3.3	3.0E-07	8.5E-05
ADORA2A	adenosine A2a receptor	3.5	1.2E-07	4.9E-05
AGPAT9	1-acylglycerol-3-phosphate O-acyltransferase 9	-2.1	3.5E-04	5.5E-03
AK4	adenylate kinase 4	3.1	3.8E-08	3.2E-05
AVPI1	arginine vasopressin-induced 1	-2.1	1.1E-04	2.7E-03
BIRC3	baculoviral IAP repeat containing 3	2.9	2.7E-07	8.1E-05
C15orf48	chromosome 15 open reading frame 48	2.6	1.3E-05	7.2E-04
C1orf162	chromosome 1 open reading frame 162	-2.8	1.0E-05	6.1E-04
CCL1	chemokine (C-C motif) ligand 1	5.0	1.2E-06	1.5E-04
CCL14	chemokine (C-C motif) ligand 14	2.2	8.9E-04	1.0E-02
CCL20	chemokine (C-C motif) ligand 20	6.2	3.1E-11	2.3E-07
CCL3	chemokine (C-C motif) ligand 3	3.3	2.1E-04	3.9E-03
CCL3L1	chemokine (C-C motif) ligand 3-like 1	4.2	1.8E-04	3.6E-03
CCL3L3	chemokine (C-C motif) ligand 3-like 3	2.1	1.3E-03	1.3E-02
CCL4L1	chemokine (C-C motif) ligand 4-like 1	4.4	5.9E-06	4.5E-04
CCL4L2	chemokine (C-C motif) ligand 4-like 2	3.7	2.0E-05	9.1E-04
CCL5	chemokine (C-C motif) ligand 5	3.3	2.8E-05	1.1E-03
CCR7	chemokine (C-C motif) receptor 7	2.8	1.1E-04	2.6E-03
CD40	CD40 molecule. TNF receptor superfamily member 5	2.1	7.7E-06	5.2E-04
CD80	CD80 molecule	2.1	7.9E-06	5.3E-04
CKB	creatine kinase. brain	3.0	3.6E-06	3.3E-04
CKLF	chemokine-like factor	-2.5	1.3E-07	5.1E-05
CSF2	colony stimulating factor 2 (granulocyte-macrophage)	4.5	3.9E-04	6.0E-03
CXCL1	chemokine (C-X-C motif) ligand 1	4.9	8.8E-09	2.0E-05
CXCL2	chemokine (C-X-C motif) ligand 2	5.4	9.3E-07	1.4E-04
CXCL8	chemokine (C-X-C motif) ligand 8	3.6	1.1E-05	6.2E-04
CYP4B1	cytochrome P450. family 4. subfamily B. polypeptide 1	2.4	8.2E-07	1.3E-04
DDIT4	DNA-damage-inducible transcript 4	2.5	1.5E-06	1.9E-04
DENND5A	DENN/MADD domain containing 5A	2.7	4.2E-05	1.4E-03
DHRS9	dehydrogenase/reductase (SDR family) member 9	-2.6	2.8E-05	1.1E-03
DNAAF1	dynein. axonemal. assembly factor 1	3.6	5.0E-07	1.0E-04

Gene	Gene Name	LogFC	P.Value	FDR
DPYSL3	dihydropyrimidinase-like 3	2.4	1.7E-03	1.6E-02
DRAM1	DNA-damage regulated autophagy modulator 1	2.2	1.8E-06	2.0E-04
DUSP5	dual specificity phosphatase 5	2.5	2.0E-05	9.1E-04
EBI3	Epstein-Barr virus induced 3	4.2	1.1E-09	5.4E-06
EHD1	EH-domain containing 1	3.8	4.0E-07	9.1E-05
ETS2	v-ets avian erythroblastosis virus E26 oncogene homolog 2	2.6	1.2E-06	1.6E-04
FGL2	fibrinogen-like 2	-2.5	1.8E-05	8.4E-04
G0S2	G0/G1 switch 2	2.5	8.4E-07	1.3E-04
GALM	galactose mutarotase (aldose 1-epimerase)	-2.4	2.0E-04	3.8E-03
GCH1	GTP cyclohydrolase 1	2.8	3.7E-06	3.4E-04
GJB2	gap junction protein. beta 2. 26kDa	3.7	2.7E-09	9.9E-06
GRAMD1A	GRAM domain containing 1A	3.7	2.3E-08	2.4E-05
GYPC	glycophorin C (Gerbich blood group)	2.1	4.5E-05	1.5E-03
HEY1	hes-related family bHLH transcription factor with YRPW motif 1	2.3	1.2E-08	2.2E-05
HLA-DMB	major histocompatibility complex. class II. DM beta	-2.2	7.0E-06	5.0E-04
ICAM1	intercellular adhesion molecule 1	2.0	4.1E-07	9.1E-05
IDO1	indoleamine 2.3-dioxygenase 1	2.6	9.6E-04	1.1E-02
IER3	immediate early response 3	3.8	3.2E-07	8.5E-05
IL1A	interleukin 1. alpha	6.2	2.1E-08	2.4E-05
IL1B	interleukin 1. beta	5.8	5.5E-07	1.1E-04
IL23A	interleukin 23. alpha subunit p19	4.7	4.5E-05	1.5E-03
IL36G	interleukin 36. gamma	3.6	4.4E-04	6.5E-03
IL6	interleukin 6	5.7	1.3E-07	5.2E-05
IL7R	interleukin 7 receptor	4.0	6.8E-08	4.2E-05
IRAK2	interleukin-1 receptor-associated kinase 2	3.0	3.4E-07	8.5E-05
IRAK3	interleukin-1 receptor-associated kinase 3	2.0	2.6E-07	8.1E-05
KYNU	kynureninase	2.4	4.7E-07	9.9E-05
LAMP3	lysosomal-associated membrane protein 3	3.9	8.0E-08	4.2E-05
LOC285628	uncharacterized LOC285628	2.0	1.0E-07	4.5E-05
LOC374443	C-type lectin domain family 2. member D pseudogene	2.1	3.7E-05	1.3E-03
LSS	lanosterol synthase (2.3-oxidosqualene-lanosterol cyclase)	2.2	3.4E-07	8.5E-05
LTA4H	leukotriene A4 hydrolase	-2.2	3.2E-04	5.3E-03
MAP3K8	mitogen-activated protein kinase kinase kinase 8	2.9	1.4E-05	7.4E-04
MARCKS	myristoylated alanine-rich protein kinase C substrate	3.6	1.2E-06	1.5E-04
MBP	myelin basic protein	-2.4	4.6E-06	3.8E-04
MCOLN2	mucolipin 2	3.8	1.3E-11	2.0E-07
MCTP1	multiple C2 domains. transmembrane 1	2.1	2.4E-08	2.4E-05
MIR155HG	MIR155 host gene (non-protein coding)	2.5	2.1E-04	3.9E-03
MIR302C	microRNA 302c	2.1	1.6E-06	1.9E-04
MSANTD3	Myb/SANT-like DNA-binding domain containing 3	2.5	4.6E-06	3.7E-04
MYO1G	myosin IG	2.0	1.1E-07	4.5E-05
NAMPT	nicotinamide phosphoribosyltransferase	2.6	5.5E-07	1.1E-04
NBN	nibrin	2.2	2.9E-07	8.5E-05
NDP	Norrie disease (pseudoglioma)	3.2	3.0E-04	5.0E-03
NFKB1	nuclear factor of kappa light polypeptide gene enhancer in B-cells 1	2.3	1.6E-05	8.0E-04

Gene	Gene Name	LogFC	P.Value	FDR
NFKBIA	nuclear factor of kappa light polypeptide gene enhancer in B-cells inhibitor. alpha	2.2	8.9E-06	5.7E-04
NFKBIZ	nuclear factor of kappa light polypeptide gene enhancer in B-cells inhibitor. zeta	3.9	9.0E-06	5.7E-04
NINJ1	ninjurin 1	2.7	7.1E-07	1.2E-04
PDE4B	phosphodiesterase 4B. cAMP-specific	2.9	2.4E-07	7.6E-05
PIM1	Pim-1 proto-oncogene. serine/threonine kinase	2.7	4.4E-06	3.7E-04
PIM2	Pim-2 proto-oncogene. serine/threonine kinase	2.8	5.9E-07	1.1E-04
PLAC8	placenta-specific 8	2.6	6.6E-04	8.5E-03
PLIN2	perilipin 2	-2.2	7.3E-05	2.1E-03
PSD3	pleckstrin and Sec7 domain containing 3	2.4	5.3E-06	4.1E-04
PSTPIP2	proline-serine-threonine phosphatase interacting protein 2	2.9	1.0E-06	1.4E-04
PTGS2	prostaglandin-endoperoxide synthase 2 (cyclooxygenase)	6.2	9.4E-09	2.0E-05
RGS2	regulator of G-protein signaling 2	-2.2	1.5E-05	7.7E-04
RHOU	ras homolog family member U	2.1	3.6E-05	1.3E-03
RIPK2	receptor-interacting serine-threonine kinase 2	2.2	1.0E-07	4.5E-05
RNF144B	ring finger protein 144B	3.1	1.9E-06	2.1E-04
SERPINB2	serpin peptidase inhibitor. clade B (ovalbumin). member 2	5.2	1.8E-05	8.4E-04
SERPINB2	serpin peptidase inhibitor. clade B (ovalbumin). member 2	5.2	1.1E-06	1.5E-04
SERPINB9	serpin peptidase inhibitor. clade B (ovalbumin). member 9	2.8	5.5E-05	1.7E-03
SLAMF1	signaling lymphocytic activation molecule family member 1	2.3	1.4E-03	1.4E-02
SLC1A2	solute carrier family 1 (glial high affinity glutamate transporter). member 2	2.2	3.4E-05	1.3E-03
SLC25A24	solute carrier family 25 (mitochondrial carrier; phosphate carrier). member 24	2.9	4.7E-06	3.8E-04
SLC2A6	solute carrier family 2 (facilitated glucose transporter). member 6	3.7	2.1E-08	2.4E-05
SLC44A2	solute carrier family 44 (choline transporter). member 2	-2.7	2.7E-07	8.1E-05
SLC7A5	solute carrier family 7 (amino acid transporter light chain. L system). member 5	2.3	8.3E-05	2.2E-03
SOD2	superoxide dismutase 2. mitochondrial	4.2	1.2E-06	1.6E-04
ST6GAL1	ST6 beta-galactosamide alpha-2.6-sialyltranferase 1	-2.3	9.2E-05	2.4E-03
STAT4	signal transducer and activator of transcription 4	3.8	1.4E-08	2.2E-05
STAT5A	signal transducer and activator of transcription 5A	2.0	1.9E-07	6.3E-05
STK26	serine/threonine protein kinase 26	3.8	8.4E-08	4.2E-05
THBS1	thrombospondin 1	3.0	1.3E-05	7.0E-04
TNF	tumor necrosis factor	5.8	3.3E-08	3.1E-05
TNFAIP3	tumor necrosis factor. alpha-induced protein 3	2.0	2.2E-06	2.4E-04
TNFAIP6	tumor necrosis factor. alpha-induced protein 6	5.3	1.7E-07	6.0E-05
TNFRSF21	tumor necrosis factor receptor superfamily. member 21	-3.2	8.1E-08	4.2E-05
TNFRSF4	tumor necrosis factor receptor superfamily. member 4	3.6	4.3E-08	3.3E-05
TNIP1	TNFAIP3 interacting protein 1	2.9	2.4E-08	2.4E-05
TNIP3	TNFAIP3 interacting protein 3	2.4	1.8E-04	3.6E-03
TRAF1	TNF receptor-associated factor 1	3.1	8.1E-09	2.0E-05
TSPAN17	tetraspanin 17	-2.2	6.3E-04	8.3E-03
UPB1	ureidopropionase. beta	2.6	8.8E-06	5.6E-04
ZC3H12A	zinc finger CCCH-type containing 12A	3.0	2.0E-07	6.6E-05
ZC3H12C	zinc finger CCCH-type containing 12C	2.5	1.2E-05	6.6E-04

List of DEGs in MoCT infected with Mtb UT205 compared to MoCT-NI.

Gene	Gene Name	LogFC	P.Value	FDR
ACP5	acid phosphatase 5. tartrate resistant	-1.8	2.9E-06	1.2E-04
ACSL1	acyl-CoA synthetase long-chain family member 1	2.1	5.2E-05	9.5E-04
ACSL5	acyl-CoA synthetase long-chain family member 5	1.8	1.6E-07	1.9E-05
ADA	adenosine deaminase	3.1	8.6E-06	2.6E-04
ADORA2A	adenosine A2a receptor	3.7	2.9E-09	1.3E-06
AK4	adenylate kinase 4	3.0	2.5E-08	5.8E-06
AKR1B1	aldo-keto reductase family 1. member B1 (aldose reductase)	2.0	3.7E-07	2.9E-05
ARHGEF6	Rac/Cdc42 guanine nucleotide exchange factor (GEF) 6	-1.8	1.4E-07	1.8E-05
ARL5B	ADP-ribosylation factor-like 5B	1.7	8.6E-06	2.6E-04
ASNS	asparagine synthetase (glutamine-hydrolyzing)	1.6	7.9E-06	2.5E-04
AVPI1	arginine vasopressin-induced 1	-1.6	4.1E-06	1.5E-04
B4GALT1	UDP-Gal:betaGlcNAc beta 1.4- galactosyltransferase. polypeptide 1	1.7	3.1E-05	6.6E-04
BASP1	brain abundant. membrane attached signal protein 1	1.7	2.9E-04	3.3E-03
BCL11A	B-cell CLL/lymphoma 11A (zinc finger protein)	1.8	3.7E-07	2.9E-05
BHLHE40	basic helix-loop-helix family. member e40	1.7	1.6E-04	2.2E-03
BIRC3	baculoviral IAP repeat containing 3	3.1	6.4E-10	4.6E-07
BTG3	BTG family. member 3	1.8	1.0E-07	1.5E-05
C15orf48	chromosome 15 open reading frame 48	2.7	3.3E-07	2.8E-05
C17orf96	chromosome 17 open reading frame 96	1.9	1.9E-05	4.7E-04
C1orf162	chromosome 1 open reading frame 162	-2.9	2.8E-06	1.2E-04
CASP1	caspase 1. apoptosis-related cysteine peptidase	1.7	5.8E-05	1.0E-03
CCDC109B	coiled-coil domain containing 109B	-1.7	3.8E-05	7.6E-04
CCL1	chemokine (C-C motif) ligand 1	4.7	7.4E-06	2.3E-04
CCL14	chemokine (C-C motif) ligand 14	2.2	5.3E-08	8.9E-06
CCL2	chemokine (C-C motif) ligand 2	1.5	7.4E-03	3.5E-02
CCL20	chemokine (C-C motif) ligand 20	6.2	3.3E-13	2.5E-09
CCL22	chemokine (C-C motif) ligand 22	2.1	3.0E-03	1.8E-02
CCL23	chemokine (C-C motif) ligand 23	2.0	9.8E-06	2.8E-04
CCL3	chemokine (C-C motif) ligand 3	3.5	8.3E-06	2.5E-04
CCL3L1	chemokine (C-C motif) ligand 3-like 1	4.5	4.0E-06	1.5E-04
CCL3L3	chemokine (C-C motif) ligand 3-like 3	2.2	1.1E-04	1.6E-03
CCL4L1	chemokine (C-C motif) ligand 4-like 1	4.6	1.2E-07	1.7E-05
CCL4L2	chemokine (C-C motif) ligand 4-like 2	3.8	6.7E-07	4.7E-05
CCL5	chemokine (C-C motif) ligand 5	3.3	4.6E-06	1.7E-04
CCL8	chemokine (C-C motif) ligand 8	2.6	2.1E-04	2.6E-03
CCR7	chemokine (C-C motif) receptor 7	3.1	5.6E-09	2.2E-06
CD1D	CD1d molecule	-1.7	4.2E-06	1.5E-04
CD40	CD40 molecule. TNF receptor superfamily member 5	2.5	2.0E-07	2.1E-05
CD44	CD44 molecule (Indian blood group)	1.5	1.4E-07	1.8E-05
CD48	CD48 molecule	1.6	3.3E-06	1.3E-04
CD80	CD80 molecule	2.0	4.9E-07	3.6E-05
CD82	CD82 molecule	1.6	4.3E-06	1.6E-04
CEBPA	CCAAT/enhancer binding protein (C/EBP). alpha	-1.8	7.7E-06	2.4E-04
CENPV	centromere protein V	-1.5	8.6E-04	7.5E-03

Gene	Gene Name	LogFC	P.Value	FDR
CFLAR	CASP8 and FADD-like apoptosis regulator	1.6	3.5E-04	3.8E-03
CKB	creatine kinase. brain	3.0	2.0E-07	2.1E-05
CKLF	chemokine-like factor	-2.2	2.5E-06	1.1E-04
CLCF1	cardiotrophin-like cytokine factor 1	2.3	1.2E-06	7.1E-05
CORO1A	coronin. actin binding protein. 1A	-1.5	2.5E-08	5.8E-06
CSF2	colony stimulating factor 2 (granulocyte-macrophage)	4.6	3.3E-07	2.8E-05
CSF2RA	colony stimulating factor 2 receptor. alpha. low-affinity (granulocyte-macrophage)	1.6	3.0E-06	1.3E-04
CSRNP1	cysteine-serine-rich nuclear protein 1	1.7	1.0E-04	1.6E-03
CXCL1	chemokine (C-X-C motif) ligand 1	5.0	3.5E-09	1.6E-06
CXCL10	chemokine (C-X-C motif) ligand 10	2.4	1.0E-02	4.5E-02
CXCL2	chemokine (C-X-C motif) ligand 2	5.2	8.7E-10	5.4E-07
CXCL8	chemokine (C-X-C motif) ligand 8	3.6	3.1E-07	2.7E-05
CYP4B1	cytochrome P450. family 4. subfamily B. polypeptide 1	2.3	2.0E-09	1.0E-06
DDIT4	DNA-damage-inducible transcript 4	3.0	1.4E-09	8.1E-07
DENND5A	DENN/MADD domain containing 5A	2.5	4.0E-05	7.9E-04
DHRS9	dehydrogenase/reductase (SDR family) member 9	-2.3	1.5E-06	8.2E-05
DNAAF1	dynein. axonemal. assembly factor 1	3.6	3.8E-08	7.6E-06
DNASE2	deoxyribonuclease II. lysosomal	-1.6	6.4E-06	2.1E-04
DOCK10	dedicator of cytokinesis 10	-2.0	2.0E-03	1.4E-02
DOPEY2	dopey family member 2	-1.9	3.4E-04	3.7E-03
DPYSL3	dihydropyrimidinase-like 3	2.2	8.1E-04	7.2E-03
DRAM1	DNA-damage regulated autophagy modulator 1	2.3	4.5E-08	8.4E-06
DUSP2	dual specificity phosphatase 2	2.2	7.9E-06	2.5E-04
DUSP5	dual specificity phosphatase 5	2.6	2.3E-07	2.3E-05
EBI3	Epstein-Barr virus induced 3	4.2	5.0E-10	3.8E-07
EHD1	EH-domain containing 1	3.7	2.0E-08	5.3E-06
ETS2	v-ets avian erythroblastosis virus E26 oncogene homolog 2	2.8	5.0E-08	8.9E-06
EVI2B	ecotropic viral integration site 2B	-1.6	6.1E-05	1.1E-03
EVL	Enah/Vasp-like	-1.9	4.9E-05	9.1E-04
F3	coagulation factor III (thromboplastin. tissue factor)	2.1	1.6E-03	1.2E-02
FAM129A	family with sequence similarity 129. member A	1.9	7.1E-06	2.3E-04
FAM188A	family with sequence similarity 188. member A	1.7	2.4E-07	2.3E-05
FAM198B	family with sequence similarity 198. member B	-1.6	4.5E-08	8.4E-06
FAM49A	family with sequence similarity 49. member A	1.5	3.0E-08	6.4E-06
FBXO38	F-box protein 38	-2.0	3.4E-06	1.3E-04
FCGR2A	Fc fragment of IgG. low affinity IIa. receptor (CD32)	1.6	8.2E-04	7.2E-03
FGL2	fibrinogen-like 2	-2.2	1.7E-05	4.4E-04
FPR2	formyl peptide receptor 2	1.6	5.3E-04	5.2E-03
FRAT2	frequently rearranged in advanced T-cell lymphomas 2	-1.6	2.2E-08	5.4E-06
FSCN1	fascin actin-bundling protein 1	1.8	2.0E-04	2.6E-03
FYB	FYN binding protein	-1.6	2.9E-03	1.8E-02
G0S2	G0/G1 switch 2	2.6	8.3E-07	5.4E-05
GALM	galactose mutarotase (aldose 1-epimerase)	-2.4	1.3E-05	3.6E-04
GBP1	guanylate binding protein 1. interferon-inducible	1.9	4.3E-05	8.3E-04
GBP2	guanylate binding protein 2. interferon-inducible	1.7	1.7E-05	4.3E-04

Gene	Gene Name	LogFC	P.Value	FDR
GBP5	guanylate binding protein 5	1.8	3.3E-03	2.0E-02
GCH1	GTP cyclohydrolase 1	3.0	2.1E-08	5.3E-06
GCLC	glutamate-cysteine ligase. catalytic subunit	-2.1	1.6E-04	2.1E-03
GJB2	gap junction protein. beta 2. 26kDa	3.8	1.6E-07	1.9E-05
GNG2	guanine nucleotide binding protein (G protein). gamma 2	1.5	3.3E-06	1.3E-04
GPC3	glypican 3	1.6	3.5E-04	3.8E-03
GPR132	G protein-coupled receptor 132	1.6	1.8E-05	4.5E-04
GPR137B	G protein-coupled receptor 137B	1.7	1.2E-06	7.0E-05
GRAMD1A	GRAM domain containing 1A	3.7	3.3E-13	2.5E-09
GYPC	glycophorin C (Gerbich blood group)	2.1	3.7E-06	1.4E-04
HBEGF	heparin-binding EGF-like growth factor	1.6	7.9E-05	1.3E-03
HCK	HCK proto-oncogene. Src family tyrosine kinase	1.9	1.8E-06	9.2E-05
HELZ2	helicase with zinc finger 2. transcriptional coactivator	1.5	9.4E-06	2.8E-04
HEY1	hes-related family bHLH transcription factor with YRPW motif 1	2.3	3.4E-05	7.0E-04
HK1	hexokinase 1	-1.6	1.1E-04	1.7E-03
HLA-DMB	major histocompatibility complex. class II. DM beta	-2.1	1.1E-06	6.6E-05
HSPB1	heat shock 27kDa protein 1	-1.8	2.7E-04	3.1E-03
ICAM1	intercellular adhesion molecule 1	2.2	8.7E-10	5.4E-07
IDH1	isocitrate dehydrogenase 1 (NADP+). soluble	-1.5	1.3E-06	7.2E-05
IDO1	indoleamine 2.3-dioxygenase 1	3.7	2.4E-05	5.5E-04
IER3	immediate early response 3	3.7	6.8E-09	2.6E-06
IFIT2	interferon-induced protein with tetratricopeptide repeats 2	1.6	5.0E-05	9.2E-04
IL1A	interleukin 1. alpha	6.5	3.3E-12	1.6E-08
IL1B	interleukin 1. beta	5.8	7.5E-09	2.7E-06
IL23A	interleukin 23. alpha subunit p19	5.3	4.4E-11	6.0E-08
IL36G	interleukin 36. gamma	3.7	8.6E-07	5.5E-05
IL6	interleukin 6	6.1	4.3E-11	6.0E-08
IL7R	interleukin 7 receptor	4.0	6.0E-07	4.3E-05
INSIG1	insulin induced gene 1	1.6	5.9E-08	9.9E-06
IRAK2	interleukin-1 receptor-associated kinase 2	3.1	2.2E-08	5.4E-06
IRAK3	interleukin-1 receptor-associated kinase 3	2.1	4.2E-08	8.0E-06
IRF1	interferon regulatory factor 1	1.5	1.1E-05	3.2E-04
ISG20	interferon stimulated exonuclease gene 20kDa	2.0	4.7E-04	4.8E-03
KCTD12	potassium channel tetramerization domain containing 12	-1.9	1.1E-06	6.6E-05
KYNU	kynureninase	2.4	1.7E-08	4.7E-06
LAMP3	lysosomal-associated membrane protein 3	3.7	4.4E-08	8.3E-06
LBR	lamin B receptor	-1.5	5.3E-08	8.9E-06
LOC285628	uncharacterized LOC285628	2.1	1.3E-05	3.5E-04
LOC374443	C-type lectin domain family 2. member D pseudogene	2.0	1.1E-07	1.6E-05
LOC606724	coronin. actin binding protein. 1A pseudogene	-1.7	2.7E-06	1.2E-04
LPAR6	lysophosphatidic acid receptor 6	-1.6	6.6E-07	4.7E-05
LSS	lanosterol synthase (2.3-oxidosqualene-lanosterol cyclase)	2.1	7.2E-08	1.1E-05
LTA4H	leukotriene A4 hydrolase	-2.3	6.1E-05	1.1E-03
MAF	v-maf avian musculoaponeurotic fibrosarcoma oncogene homolog	-1.5	1.9E-06	9.6E-05
MAFF	v-maf avian musculoaponeurotic fibrosarcoma oncogene homolog F	2.0	2.6E-08	5.8E-06

Gene	Gene Name	LogFC	P.Value	FDR
MAP3K4	mitogen-activated protein kinase kinase kinase 4	2.0	3.8E-06	1.4E-04
MAP3K8	mitogen-activated protein kinase kinase kinase 8	3.0	1.9E-07	2.1E-05
MARCKS	myristoylated alanine-rich protein kinase C substrate	3.6	2.2E-08	5.4E-06
MB21D2	Mab-21 domain containing 2	2.1	6.8E-07	4.7E-05
MBP	myelin basic protein	-2.2	4.0E-06	1.5E-04
MCOLN2	mucolipin 2	3.9	1.0E-08	3.3E-06
MCTP1	multiple C2 domains. transmembrane 1	1.9	1.9E-11	4.6E-08
MERTK	MER proto-oncogene. tyrosine kinase	-1.6	4.0E-06	1.5E-04
MIR155HG	MIR155 host gene (non-protein coding)	2.7	5.4E-06	1.8E-04
MIR302C	microRNA 302c	2.1	2.2E-07	2.3E-05
MNDA	myeloid cell nuclear differentiation antigen	-1.9	1.1E-07	1.6E-05
MSANTD3	Myb/SANT-like DNA-binding domain containing 3	2.5	3.0E-08	6.4E-06
MSC	musculin	1.7	1.5E-06	8.2E-05
MT2A	metallothionein 2A	1.6	2.0E-03	1.4E-02
MX1	MX dynamin-like GTPase 1	1.5	2.0E-03	1.4E-02
MYO1G	myosin IG	1.9	2.4E-07	2.3E-05
NAMPT	nicotinamide phosphoribosyltransferase	2.9	1.4E-09	8.1E-07
NBN	nibrin	2.4	7.3E-09	2.7E-06
NCEH1	neutral cholesterol ester hydrolase 1	-1.7	1.0E-08	3.3E-06
NDP	Norrie disease (pseudoglioma)	3.4	3.4E-06	1.3E-04
NFKB1	nuclear factor of kappa light polypeptide gene enhancer in B-cells 1	2.3	2.4E-06	1.1E-04
NFKBIA	nuclear factor of kappa light polypeptide gene enhancer in B-cells inhibitor. alpha	2.3	2.8E-07	2.6E-05
NFKBIZ	nuclear factor of kappa light polypeptide gene enhancer in B-cells inhibitor. zeta	3.7	2.0E-06	1.0E-04
NINJ1	ninjurin 1	2.5	1.6E-07	1.9E-05
OSM	oncostatin M	1.7	1.6E-05	4.1E-04
P2RX7	purinergic receptor P2X. ligand-gated ion channel. 7	1.7	1.5E-04	2.1E-03
PDE4B	phosphodiesterase 4B. cAMP-specific	3.4	1.4E-10	1.5E-07
PDK4	pyruvate dehydrogenase kinase. isozyme 4	-1.8	2.0E-07	2.1E-05
PDSS1	prenyl (decaprenyl) diphosphate synthase. subunit 1	1.5	2.9E-04	3.3E-03
PECAM1	platelet/endothelial cell adhesion molecule 1	-1.7	5.7E-05	1.0E-03
PFKFB3	6-phosphofructo-2-kinase/fructose-2.6-biphosphatase 3	1.6	3.4E-05	6.9E-04
PFKFB4	6-phosphofructo-2-kinase/fructose-2.6-biphosphatase 4	-1.7	7.1E-06	2.3E-04
PILRA	paired immunoglobin-like type 2 receptor alpha	1.8	4.6E-07	3.5E-05
PIM1	Pim-1 proto-oncogene. serine/threonine kinase	3.1	1.4E-08	4.2E-06
PIM2	Pim-2 proto-oncogene. serine/threonine kinase	2.9	2.9E-10	2.7E-07
PLAC8	placenta-specific 8	2.3	1.8E-04	2.3E-03
PLAUR	plasminogen activator. urokinase receptor	1.8	1.5E-06	8.1E-05
PLIN2	perilipin 2	-2.4	2.4E-09	1.1E-06
PLXDC2	plexin domain containing 2	-1.8	2.2E-06	1.0E-04
PPARG	peroxisome proliferator-activated receptor gamma	-1.8	3.7E-07	2.9E-05
PPP3CC	protein phosphatase 3. catalytic subunit. gamma isozyme	1.8	2.8E-06	1.2E-04
PSD3	pleckstrin and Sec7 domain containing 3	2.5	1.8E-07	2.0E-05
PSTPIP2	proline-serine-threonine phosphatase interacting protein 2	2.8	1.8E-07	2.0E-05
PTGER4	prostaglandin E receptor 4 (subtype EP4)	1.5	3.1E-05	6.5E-04
PTGS2	prostaglandin-endoperoxide synthase 2 (cyclooxygenase)	6.4	1.5E-11	4.6E-08

Gene	Gene Name	LogFC	P.Value	FDR
PTPRO	protein tyrosine phosphatase. receptor type. O	-1.7	2.4E-06	1.1E-04
PYCARD	PYD and CARD domain containing	-1.5	2.6E-06	1.1E-04
RAPGEF2	Rap guanine nucleotide exchange factor (GEF) 2	1.6	2.1E-03	1.4E-02
RCBTB2	regulator of chromosome condensation (RCC1) and BTB (POZ) domain containing protein 2	-1.7	1.3E-06	7.4E-05
REL	v-rel avian reticuloendotheliosis viral oncogene homolog	1.5	1.5E-03	1.1E-02
RGS1	regulator of G-protein signaling 1	-1.8	2.1E-05	4.9E-04
RGS16	regulator of G-protein signaling 16	1.8	6.6E-05	1.1E-03
RGS2	regulator of G-protein signaling 2	-2.2	1.7E-06	8.9E-05
RHOBTB3	Rho-related BTB domain containing 3	1.5	1.9E-03	1.3E-02
RHOU	ras homolog family member U	2.0	5.2E-06	1.8E-04
RIPK2	receptor-interacting serine-threonine kinase 2	2.5	3.7E-10	3.2E-07
RNF144B	ring finger protein 144B	3.3	2.5E-08	5.8E-06
RNF19B	ring finger protein 19B	1.6	3.3E-06	1.3E-04
SAMSN1	SAM domain. SH3 domain and nuclear localization signals 1	2.0	2.0E-04	2.6E-03
SDC4	syndecan 4	1.8	1.8E-05	4.5E-04
SDS	serine dehydratase	-1.6	9.0E-06	2.7E-04
SERPINB2	serpin peptidase inhibitor. clade B (ovalbumin). member 2	5.3	3.8E-07	3.0E-05
SERPINB9	serpin peptidase inhibitor. clade B (ovalbumin). member 9	2.8	3.9E-07	3.0E-05
SLAMF1	signaling lymphocytic activation molecule family member 1	2.7	2.3E-05	5.3E-04
SLAMF7	SLAM family member 7	1.9	5.3E-07	3.9E-05
SLC16A6	solute carrier family 16. member 6	1.6	5.6E-04	5.5E-03
SLC1A2	solute carrier family 1 (glial high affinity glutamate transporter). member 2	2.2	5.6E-06	1.9E-04
SLC25A24	solute carrier family 25 (mitochondrial carrier; phosphate carrier). member 24	2.8	1.1E-07	1.6E-05
SLC2A3	solute carrier family 2 (facilitated glucose transporter). member 3	2.0	5.7E-07	4.2E-05
SLC2A6	solute carrier family 2 (facilitated glucose transporter). member 6	3.7	2.4E-11	4.6E-08
SLC39A8	solute carrier family 39 (zinc transporter). member 8	1.5	5.1E-04	5.1E-03
SLC44A2	solute carrier family 44 (choline transporter). member 2	-2.3	1.4E-07	1.8E-05
SLC7A11	solute carrier family 7 (anionic amino acid transporter light chain. xc- system). member 11	1.8	1.9E-03	1.3E-02
SLC7A5	solute carrier family 7 (amino acid transporter light chain. L system). member 5	2.3	4.2E-06	1.5E-04
SMS	spermine synthase	1.6	3.1E-07	2.7E-05
SNHG15	small nucleolar RNA host gene 15 (non-protein coding)	1.8	4.3E-07	3.4E-05
SOCS3	suppressor of cytokine signaling 3	2.5	4.6E-08	8.4E-06
SOD2	superoxide dismutase 2. mitochondrial	4.2	8.0E-09	2.8E-06
ST6GAL1	ST6 beta-galactosamide alpha-2.6-sialyltranferase 1	-2.1	2.9E-04	3.3E-03
STAT4	signal transducer and activator of transcription 4	3.8	1.2E-08	3.6E-06
STAT5A	signal transducer and activator of transcription 5A	2.0	1.7E-08	4.6E-06
STK26	serine/threonine protein kinase 26	3.7	2.0E-09	1.0E-06
STX11	syntaxin 11	1.5	2.5E-07	2.4E-05
TEX2	testis expressed 2	-1.8	2.4E-06	1.1E-04
TGFBI	transforming growth factor. beta-induced. 68kDa	-1.6	7.5E-04	6.8E-03
THBS1	thrombospondin 1	2.5	5.0E-03	2.7E-02
TIMP2	TIMP metallopeptidase inhibitor 2	-1.7	8.8E-05	1.4E-03
TM6SF1	transmembrane 6 superfamily member 1	-1.7	1.6E-08	4.5E-06
TMEM194A	transmembrane protein 194A	1.7	2.1E-05	5.0E-04
TNF	tumor necrosis factor	6.0	2.4E-11	4.6E-08

Gene	Gene Name	LogFC	P.Value	FDR
TNFAIP2	tumor necrosis factor. alpha-induced protein 2	1.7	6.5E-07	4.6E-05
TNFAIP3	tumor necrosis factor. alpha-induced protein 3	2.0	6.9E-07	4.7E-05
TNFAIP6	tumor necrosis factor. alpha-induced protein 6	5.6	4.8E-10	3.8E-07
TNFAIP8	tumor necrosis factor. alpha-induced protein 8	1.8	3.6E-06	1.4E-04
TNFAIP8L3	tumor necrosis factor. alpha-induced protein 8-like 3	-1.8	1.2E-07	1.6E-05
TNFRSF21	tumor necrosis factor receptor superfamily. member 21	-2.8	4.9E-06	1.7E-04
TNFRSF4	tumor necrosis factor receptor superfamily. member 4	3.6	4.3E-09	1.9E-06
TNFRSF9	tumor necrosis factor receptor superfamily. member 9	1.5	1.1E-06	6.4E-05
TNIP1	TNFAIP3 interacting protein 1	2.8	4.3E-09	1.9E-06
TNIP3	TNFAIP3 interacting protein 3	2.6	2.1E-06	1.0E-04
TRAF1	TNF receptor-associated factor 1	3.1	5.6E-09	2.2E-06
TREM2	triggering receptor expressed on myeloid cells 2	-1.8	9.1E-05	1.4E-03
TSPAN17	tetraspanin 17	-1.8	2.1E-03	1.4E-02
TSPAN33	tetraspanin 33	2.0	5.0E-04	5.0E-03
TXN	thioredoxin	1.7	3.8E-05	7.5E-04
UBASH3B	ubiquitin associated and SH3 domain containing B	-1.9	9.6E-06	2.8E-04
UPB1	ureidopropionase. beta	2.5	2.6E-08	5.8E-06
USP12	ubiquitin specific peptidase 12	1.7	1.5E-09	8.3E-07
VAV3	vav 3 guanine nucleotide exchange factor	-1.7	3.6E-04	3.9E-03
VEGFB	vascular endothelial growth factor B	-1.9	5.9E-06	2.0E-04
WTAP	Wilms tumor 1 associated protein	1.9	2.2E-06	1.0E-04
ZC3H12A	zinc finger CCCH-type containing 12A	2.7	2.0E-07	2.1E-05
ZC3H12C	zinc finger CCCH-type containing 12C	2.5	5.9E-07	4.3E-05
ZP3	zona pellucida glycoprotein 3 (sperm receptor)	1.8	4.8E-04	4.9E-03
ZSWIM4	zinc finger. SWIM-type containing 4	1.5	6.4E-06	2.1E-04

List of DEGs in MoTB infected with Mtb UT127 compared to MoTB-NI.

Gene	Gene Name	LogFC	P.Value	FDR
ACSL1	acyl-CoA synthetase long-chain family member 1	1.8	6.3E-05	1.1E-02
ADA	adenosine deaminase	3.7	5.8E-04	2.4E-02
ADORA2A	adenosine A2a receptor	3.1	1.4E-03	3.4E-02
AGPAT9	1-acylglycerol-3-phosphate O-acyltransferase 9	-2.2	1.2E-04	1.3E-02
AK4	adenylate kinase 4	1.9	5.3E-05	1.0E-02
ARHGEF3	Rho guanine nucleotide exchange factor (GEF) 3	-1.6	4.8E-05	9.9E-03
ARHGEF6	Rac/Cdc42 guanine nucleotide exchange factor (GEF) 6	-1.8	1.1E-05	6.9E-03
ATP2B4	ATPase. Ca++ transporting. plasma membrane 4	-2.3	2.5E-03	4.6E-02
B4GALT1	UDP-Gal:betaGlcNAc beta 1.4- galactosyltransferase. polypeptide 1	1.7	9.9E-04	3.0E-02
BCL11A	B-cell CLL/lymphoma 11A (zinc finger protein)	2.0	2.3E-03	4.4E-02
BIRC3	baculoviral IAP repeat containing 3	2.7	9.1E-04	2.9E-02
BTG3	BTG family. member 3	1.5	8.1E-06	5.9E-03
C15orf48	chromosome 15 open reading frame 48	2.3	6.8E-04	2.5E-02
C1orf162	chromosome 1 open reading frame 162	-2.6	8.6E-05	1.2E-02
CASP1	caspase 1. apoptosis-related cysteine peptidase	1.8	1.4E-04	1.4E-02
CCL14	chemokine (C-C motif) ligand 14	1.6	1.6E-03	3.8E-02

Gene	Gene Name	LogFC	P.Value	FDR
CCND2	cyclin D2	-2.6	1.7E-04	1.5E-02
CEBPA	CCAAT/enhancer binding protein (C/EBP). alpha	-1.8	1.2E-04	1.3E-02
CHD9	chromodomain helicase DNA binding protein 9	-1.8	1.6E-04	1.5E-02
CKB	creatine kinase. brain	3.5	1.7E-03	3.9E-02
CKLF	chemokine-like factor	-2.4	7.1E-05	1.1E-02
CORO2A	coronin. actin binding protein. 2A	-1.6	1.7E-05	7.8E-03
CSF1R	colony stimulating factor 1 receptor	-1.6	1.4E-04	1.4E-02
DCSTAMP	dendrocyte expressed seven transmembrane protein	-2.0	4.2E-05	9.6E-03
DDIT4	DNA-damage-inducible transcript 4	2.1	4.6E-04	2.2E-02
DENND5A	DENN/MADD domain containing 5A	2.4	2.3E-03	4.4E-02
DHRS3	dehydrogenase/reductase (SDR family) member 3	-1.6	4.2E-04	2.1E-02
DHRS9	dehydrogenase/reductase (SDR family) member 9	-3.0	2.7E-04	1.8E-02
DNAAF1	dynein. axonemal. assembly factor 1	2.9	5.9E-04	2.4E-02
DOCK10	dedicator of cytokinesis 10	-2.4	4.2E-04	2.1E-02
DOPEY2	dopey family member 2	-2.0	2.7E-04	1.8E-02
DRAM1	DNA-damage regulated autophagy modulator 1	1.9	1.5E-03	3.6E-02
EGR2	early growth response 2	-1.6	9.9E-04	3.0E-02
EMP1	epithelial membrane protein 1	-1.9	1.8E-04	1.5E-02
EVI2B	ecotropic viral integration site 2B	-1.7	2.2E-04	1.6E-02
EVL	Enah/Vasp-like	-2.4	3.3E-05	9.5E-03
FAM49A	family with sequence similarity 49. member A	1.8	2.9E-04	1.8E-02
FANCE	Fanconi anemia. complementation group E	-1.7	5.4E-04	2.3E-02
FBXO38	F-box protein 38	-1.9	1.1E-04	1.3E-02
FHL1	four and a half LIM domains 1	-1.7	1.5E-03	3.6E-02
FOS	FBJ murine osteosarcoma viral oncogene homolog	-1.8	2.7E-04	1.8E-02
GALM	galactose mutarotase (aldose 1-epimerase)	-2.2	5.7E-06	5.9E-03
GCH1	GTP cyclohydrolase 1	2.1	9.1E-04	2.9E-02
GCLC	glutamate-cysteine ligase. catalytic subunit	-2.0	3.6E-04	1.9E-02
GFOD1	glucose-fructose oxidoreductase domain containing 1	-1.6	1.1E-04	1.3E-02
GJB2	gap junction protein. beta 2. 26kDa	3.9	4.5E-04	2.2E-02
GPC3	glypican 3	1.6	2.2E-04	1.6E-02
GPR137B	G protein-coupled receptor 137B	1.6	8.1E-04	2.8E-02
GRAMD1A	GRAM domain containing 1A	3.1	2.4E-03	4.5E-02
GYPC	glycophorin C (Gerbich blood group)	2.1	2.4E-04	1.6E-02
HCK	HCK proto-oncogene. Src family tyrosine kinase	2.1	2.5E-03	4.6E-02
HEY1	hes-related family bHLH transcription factor with YRPW motif 1	1.9	3.2E-04	1.9E-02
HK3	hexokinase 3 (white cell)	-1.5	2.8E-03	4.9E-02
HLA-DMB	major histocompatibility complex. class II. DM beta	-2.1	1.4E-04	1.4E-02
HPCAL1	hippocalcin-like 1	-1.7	1.0E-03	3.1E-02
IDH1	isocitrate dehydrogenase 1 (NADP+). soluble	-1.8	8.1E-05	1.2E-02
IL7R	interleukin 7 receptor	3.8	6.1E-04	2.4E-02
ITGB8	integrin. beta 8	1.8	1.2E-04	1.3E-02
KCTD12	potassium channel tetramerization domain containing 12	-1.5	6.5E-04	2.5E-02
KYNU	kynureninase	1.6	1.7E-03	3.9E-02
LAD1	ladinin 1	1.6	3.4E-04	1.9E-02

Gene	Gene Name	LogFC	P.Value	FDR
LOC374443	C-type lectin domain family 2. member D pseudogene	1.9	6.9E-05	1.1E-02
LOC606724	coronin. actin binding protein. 1A pseudogene	-1.7	3.6E-06	5.9E-03
LSS	lanosterol synthase (2.3-oxidosqualene-lanosterol cyclase)	1.9	7.2E-04	2.6E-02
LTA4H	leukotriene A4 hydrolase	-2.1	1.0E-03	3.1E-02
MAP1LC3A	microtubule-associated protein 1 light chain 3 alpha	1.7	1.2E-04	1.3E-02
MAP3K8	mitogen-activated protein kinase kinase kinase 8	2.6	3.4E-04	1.9E-02
MBP	myelin basic protein	-2.3	2.1E-04	1.6E-02
MCOLN2	mucolipin 2	2.9	2.5E-03	4.7E-02
MCTP1	multiple C2 domains. transmembrane 1	1.9	1.2E-04	1.3E-02
MERTK	MER proto-oncogene. tyrosine kinase	-1.8	1.2E-05	7.2E-03
METTL7B	methyltransferase like 7B	-1.7	1.7E-03	3.8E-02
MNDA	myeloid cell nuclear differentiation antigen	-1.7	4.5E-05	9.8E-03
MSANTD3	Myb/SANT-like DNA-binding domain containing 3	2.0	1.2E-04	1.3E-02
MYO1G	myosin IG	2.2	1.8E-03	3.9E-02
NAMPT	nicotinamide phosphoribosyltransferase	2.8	1.8E-03	4.0E-02
NBN	nibrin	2.2	9.8E-04	3.0E-02
NCK2	NCK adaptor protein 2	1.6	1.1E-06	5.9E-03
NDP	Norrie disease (pseudoglioma)	2.3	2.3E-03	4.4E-02
NFKB1	nuclear factor of kappa light polypeptide gene enhancer in B-cells 1	2.1	2.0E-03	4.2E-02
NINJ1	ninjurin 1	2.2	6.2E-04	2.4E-02
OGFRL1	opioid growth factor receptor-like 1	1.8	8.9E-04	2.9E-02
PACSIN2	protein kinase C and casein kinase substrate in neurons 2	-1.5	1.0E-04	1.3E-02
PDE4B	phosphodiesterase 4B. cAMP-specific	2.4	1.3E-03	3.4E-02
PECAM1	platelet/endothelial cell adhesion molecule 1	-1.6	1.3E-03	3.3E-02
PFKFB3	6-phosphofructo-2-kinase/fructose-2.6-biphosphatase 3	1.7	2.1E-03	4.3E-02
PFKFB4	6-phosphofructo-2-kinase/fructose-2.6-biphosphatase 4	-2.2	2.1E-05	7.8E-03
PIM2	Pim-2 proto-oncogene. serine/threonine kinase	2.3	4.1E-04	2.1E-02
PLAC8	placenta-specific 8	2.8	5.3E-06	5.9E-03
PLXDC2	plexin domain containing 2	-1.8	5.4E-05	1.0E-02
PPARG	peroxisome proliferator-activated receptor gamma	-2.0	1.7E-04	1.5E-02
PSD3	pleckstrin and Sec7 domain containing 3	2.2	7.8E-04	2.7E-02
PSTPIP2	proline-serine-threonine phosphatase interacting protein 2	2.6	1.2E-04	1.3E-02
PTPRO	protein tyrosine phosphatase. receptor type. O	-1.6	3.2E-04	1.9E-02
PYCARD	PYD and CARD domain containing	-2.0	1.1E-04	1.3E-02
RAB11FIP1	RAB11 family interacting protein 1 (class I)	-1.6	5.3E-05	1.0E-02
RARRES1	retinoic acid receptor responder (tazarotene induced) 1	1.8	3.5E-05	9.5E-03
RCBTB2	regulator of chromosome condensation (RCC1) and BTB (POZ) domain containing protein 2	-2.0	1.2E-04	1.3E-02
RCN1	reticulocalbin 1. EF-hand calcium binding domain	1.5	3.5E-04	1.9E-02
RDX	radixin	1.6	1.0E-03	3.1E-02
RIPK2	receptor-interacting serine-threonine kinase 2	1.9	2.6E-03	4.7E-02
RNF144B	ring finger protein 144B	2.4	9.3E-05	1.2E-02
SAMSN1	SAM domain. SH3 domain and nuclear localization signals 1	1.7	2.2E-04	1.6E-02
SERPINB9	serpin peptidase inhibitor. clade B (ovalbumin). member 9	2.2	1.1E-03	3.1E-02
SGK223	homolog of rat pragma of Rnd2	-1.7	3.5E-04	1.9E-02
SHPK	sedoheptulokinase	-1.6	4.0E-04	2.0E-02

Gene	Gene Name	LogFC	P.Value	FDR
SLAMF7	SLAM family member 7	2.0	1.7E-03	3.8E-02
SLC1A2	solute carrier family 1 (glial high affinity glutamate transporter). member 2	2.7	2.8E-04	1.8E-02
SLC27A3	solute carrier family 27 (fatty acid transporter). member 3	-1.5	2.8E-06	5.9E-03
SLC2A6	solute carrier family 2 (facilitated glucose transporter). member 6	3.2	1.0E-03	3.1E-02
SLC30A3	solute carrier family 30 (zinc transporter). member 3	-2.3	1.1E-03	3.1E-02
SLC43A2	solute carrier family 43 (amino acid system L transporter). member 2	1.6	1.6E-03	3.8E-02
SLC44A2	solute carrier family 44 (choline transporter). member 2	-2.6	5.1E-05	1.0E-02
SLCO2B1	solute carrier organic anion transporter family. member 2B1	-1.8	1.1E-04	1.3E-02
SLFN11	schlafen family member 11	-1.5	8.8E-05	1.2E-02
SMS	spermine synthase	1.6	7.5E-05	1.2E-02
SNHG15	small nucleolar RNA host gene 15 (non-protein coding)	1.8	4.5E-06	5.9E-03
SOCS3	suppressor of cytokine signaling 3	2.2	1.3E-03	3.3E-02
SOD2	superoxide dismutase 2. mitochondrial	3.8	1.6E-03	3.8E-02
SORT1	sortilin 1	-1.7	6.3E-05	1.1E-02
SPRED1	sprouty-related. EVH1 domain containing 1	-1.6	1.2E-03	3.2E-02
SPRY2	sprouty homolog 2 (Drosophila)	-1.9	1.0E-03	3.1E-02
ST6GAL1	ST6 beta-galactosamide alpha-2.6-sialyltranferase 1	-2.7	1.2E-03	3.2E-02
STAT4	signal transducer and activator of transcription 4	3.4	7.8E-04	2.7E-02
STK26	serine/threonine protein kinase 26	3.0	2.2E-04	1.6E-02
TACSTD2	tumor-associated calcium signal transducer 2	-1.6	4.2E-04	2.1E-02
TBC1D10C	TBC1 domain family. member 10C	-1.6	5.4E-05	1.0E-02
TEX2	testis expressed 2	-1.5	1.2E-04	1.3E-02
TIMP2	TIMP metallopeptidase inhibitor 2	-1.7	1.4E-04	1.4E-02
TM6SF1	transmembrane 6 superfamily member 1	-1.6	9.2E-05	1.2E-02
TNFRSF21	tumor necrosis factor receptor superfamily. member 21	-3.4	4.5E-05	9.8E-03
TNFRSF4	tumor necrosis factor receptor superfamily. member 4	2.8	1.1E-03	3.2E-02
TNIP1	TNFAIP3 interacting protein 1	2.5	2.8E-03	4.9E-02
TNIP3	TNFAIP3 interacting protein 3	2.6	3.7E-04	1.9E-02
TRAF1	TNF receptor-associated factor 1	2.5	1.6E-03	3.8E-02
TSPAN17	tetraspanin 17	-2.7	5.5E-04	2.3E-02
UBASH3B	ubiquitin associated and SH3 domain containing B	-1.6	2.1E-04	1.6E-02
UPB1	ureidopropionase. beta	2.0	2.1E-04	1.6E-02
VCL	vinculin	-1.6	7.5E-04	2.6E-02
WTAP	Wilms tumor 1 associated protein	1.7	1.4E-04	1.4E-02
ZC3H12C	zinc finger CCCH-type containing 12C	1.8	3.3E-04	1.9E-02
ZP3	zona pellucida glycoprotein 3 (sperm receptor)	2.1	1.5E-04	1.4E-02

List of DEGs in MoTB infected with Mtb UT205 compared to MoTB-NI.

Gene	Gene Name	LogFC	P.Value	FDR
ACSL1	acyl-CoA synthetase long-chain family member 1	1.8	1.3E-04	1.7E-02
ADA	adenosine deaminase	3.5	5.1E-04	2.9E-02
ADORA2A	adenosine A2a receptor	2.8	9.0E-04	3.6E-02
AK4	adenylate kinase 4	1.9	2.4E-04	2.2E-02
BASP1	brain abundant. membrane attached signal protein 1	1.8	2.9E-04	2.4E-02

Gene	Gene Name	LogFC	P.Value	FDR
BCL11A	B-cell CLL/lymphoma 11A (zinc finger protein)	1.6	8.1E-04	3.4E-02
BIRC3	baculoviral IAP repeat containing 3	2.5	4.6E-04	2.8E-02
C15orf48	chromosome 15 open reading frame 48	2.3	3.6E-04	2.6E-02
C1orf162	chromosome 1 open reading frame 162	-1.8	1.2E-03	4.2E-02
CASP1	caspase 1. apoptosis-related cysteine peptidase	1.6	4.2E-05	1.3E-02
CCL5	chemokine (C-C motif) ligand 5	1.5	1.2E-03	4.2E-02
CCND2	cyclin D2	-2.2	1.1E-05	9.8E-03
CCR7	chemokine (C-C motif) receptor 7	2.8	1.1E-03	4.0E-02
CEBPA	CCAAT/enhancer binding protein (C/EBP). alpha	-1.5	6.6E-04	3.2E-02
CKB	creatine kinase. brain	2.9	2.4E-04	2.1E-02
DCSTAMP	dendrocyte expressed seven transmembrane protein	-1.6	6.5E-04	3.2E-02
DDIT4	DNA-damage-inducible transcript 4	2.1	8.1E-05	1.4E-02
DENND5A	DENN/MADD domain containing 5A	2.0	2.0E-03	4.9E-02
DNAAF1	dynein. axonemal. assembly factor 1	2.6	3.1E-04	2.4E-02
DRAM1	DNA-damage regulated autophagy modulator 1	1.8	1.0E-03	3.8E-02
EGR2	early growth response 2	-1.6	4.4E-04	2.8E-02
EMP1	epithelial membrane protein 1	-1.6	3.9E-05	1.3E-02
FAM49A	family with sequence similarity 49. member A	1.7	2.1E-06	8.4E-03
FOS	FBJ murine osteosarcoma viral oncogene homolog	-1.6	1.7E-04	1.9E-02
G0S2	G0/G1 switch 2	2.6	3.2E-04	2.5E-02
GCH1	GTP cyclohydrolase 1	2.4	5.0E-04	2.9E-02
GJB2	gap junction protein. beta 2. 26kDa	3.7	5.0E-04	2.9E-02
GRAMD1A	GRAM domain containing 1A	2.8	1.3E-03	4.3E-02
GREM1	gremlin 1. DAN family BMP antagonist	-1.9	4.3E-04	2.8E-02
GYPC	glycophorin C (Gerbich blood group)	2.0	1.1E-04	1.6E-02
IL36G	interleukin 36. gamma	2.0	1.7E-03	4.8E-02
IL7R	interleukin 7 receptor	3.6	4.5E-04	2.8E-02
KYNU	kynureninase	1.8	1.7E-03	4.7E-02
LOC374443	C-type lectin domain family 2. member D pseudogene	1.7	1.5E-04	1.8E-02
MAP1LC3A	microtubule-associated protein 1 light chain 3 alpha	1.6	7.2E-05	1.4E-02
MAP3K8	mitogen-activated protein kinase kinase kinase 8	2.3	1.6E-03	4.6E-02
MBP	myelin basic protein	-1.8	1.9E-03	4.9E-02
MCOLN2	mucolipin 2	2.8	1.7E-03	4.8E-02
MCTP1	multiple C2 domains. transmembrane 1	1.6	8.4E-05	1.4E-02
MSANTD3	Myb/SANT-like DNA-binding domain containing 3	1.6	6.9E-05	1.4E-02
MYO1G	myosin IG	1.8	8.7E-04	3.5E-02
NAMPT	nicotinamide phosphoribosyltransferase	2.6	1.0E-03	3.8E-02
NBN	nibrin	2.1	7.8E-05	1.4E-02
NFKB1	nuclear factor of kappa light polypeptide gene enhancer in B-cells 1	2.0	1.4E-03	4.3E-02
NINJ1	ninjurin 1	2.0	6.0E-04	3.1E-02
PDE4B	phosphodiesterase 4B. cAMP-specific	2.7	2.1E-04	2.1E-02
PFKFB3	6-phosphofructo-2-kinase/fructose-2.6-biphosphatase 3	1.7	7.3E-04	3.3E-02
PILRA	paired immunoglobin-like type 2 receptor alpha	1.5	7.4E-04	3.3E-02
PIM1	Pim-1 proto-oncogene. serine/threonine kinase	1.6	1.9E-03	4.9E-02
PIM2	Pim-2 proto-oncogene. serine/threonine kinase	2.1	9.5E-05	1.5E-02

Gene	Gene Name	LogFC	P.Value	FDR
PLAC8	placenta-specific 8	2.7	5.0E-05	1.3E-02
PPARG	peroxisome proliferator-activated receptor gamma	-1.8	3.4E-05	1.3E-02
PSD3	pleckstrin and Sec7 domain containing 3	1.9	4.7E-04	2.8E-02
PSTPIP2	proline-serine-threonine phosphatase interacting protein 2	2.5	1.2E-04	1.7E-02
RCBTB2	regulator of chromosome condensation (RCC1) and BTB (POZ) domain containing protein 2	-1.5	3.8E-04	2.6E-02
RGS1	regulator of G-protein signaling 1	-1.5	4.1E-04	2.7E-02
RIPK2	receptor-interacting serine-threonine kinase 2	1.8	1.7E-04	1.9E-02
RNF144B	ring finger protein 144B	2.3	1.4E-04	1.7E-02
SAMSN1	SAM domain. SH3 domain and nuclear localization signals 1	1.8	3.8E-04	2.6E-02
SERPINB9	serpin peptidase inhibitor. clade B (ovalbumin). member 9	2.1	3.4E-04	2.6E-02
SGK223	homolog of rat pragma of Rnd2	-1.6	5.2E-04	2.9E-02
SLAMF7	SLAM family member 7	1.7	2.0E-03	4.9E-02
SLC1A2	solute carrier family 1 (glial high affinity glutamate transporter). member 2	2.3	9.9E-05	1.5E-02
SLC2A6	solute carrier family 2 (facilitated glucose transporter). member 6	2.9	1.6E-03	4.6E-02
SLC43A2	solute carrier family 43 (amino acid system L transporter). member 2	1.6	1.4E-03	4.4E-02
SOCS3	suppressor of cytokine signaling 3	2.0	1.0E-04	1.5E-02
SOD2	superoxide dismutase 2. mitochondrial	3.6	2.0E-03	4.9E-02
SPRY2	sprouty homolog 2 (Drosophila)	-1.7	6.6E-05	1.4E-02
STAT4	signal transducer and activator of transcription 4	3.2	4.9E-04	2.8E-02
STK26	serine/threonine protein kinase 26	2.7	3.0E-04	2.4E-02
TNFAIP2	tumor necrosis factor. alpha-induced protein 2	1.6	3.8E-04	2.6E-02
TNFRSF21	tumor necrosis factor receptor superfamily. member 21	-2.6	1.1E-05	9.8E-03
TNFRSF4	tumor necrosis factor receptor superfamily. member 4	2.6	8.1E-04	3.4E-02
TNIP3	TNFAIP3 interacting protein 3	2.5	5.2E-05	1.3E-02
TRAF1	TNF receptor-associated factor 1	2.3	1.2E-03	4.2E-02
TSPAN33	tetraspanin 33	1.9	1.3E-03	4.2E-02
UPB1	ureidopropionase. beta	1.8	7.4E-05	1.4E-02
WTAP	Wilms tumor 1 associated protein	1.7	1.3E-04	1.7E-02
ZP3	zona pellucida glycoprotein 3 (sperm receptor)	2.0	2.6E-04	2.2E-02
