# Supplementary material for: Infection of Monocytes From Tuberculosis Patients With Two Virulent Clinical Isolates of Mycobacterium tuberculosis Induces Alterations in Myeloid Effector Functions
Source: Front Cell Infect Microbiol. 2020 Apr 23;10:163. doi: 10.3389/fcimb.2020.00163 (PMC7190864; doi:10.3389/fcimb.2020.00163)
Supplement: Supplementary file 2 [file Data_Sheet_2.zip › Table S4.pdf]

Supplementary table 4: Analysis comparing the list of DEGs expressed between MoCT and MoTB infected with the clinical isolates of Mtb UT127 and UT205. Up-regulated genes and down-regulated genes are highlighted in red and green respectively.

Genes	MoCT-127	MoCT-205	MoTB-127	MoTB-205
Core transcriptome Monocytes-Mtb (78 genes)				
ACSL1	2.2	2.1	1.8	1.8
ADA	3.3	3.1	3.7	3.5
ADORA2A	3.5	3.7	3.1	2.8
AK4	3.1	3.0	1.9	1.9
BIRC3	2.9	3.1	2.7	2.5
C15orf48	2.6	2.7	2.3	2.3
C1orf162	-2.8	-2.9	-2.6	-1.8
CKB	3.0	3.0	3.5	2.9
DDIT4	2.5	3.0	2.1	2.1
DENND5A	2.7	2.5	2.4	2.0
DNAAF1	3.6	3.6	2.9	2.6
DRAM1	2.2	2.3	1.9	1.8
GCH1	2.8	3.0	2.1	2.4
GJB2	3.7	3.8	3.9	3.7
GRAMD1A	3.7	3.7	3.1	2.8
GYPC	2.1	2.1	2.1	2.0
IL7R	4.0	4.0	3.8	3.6
KYNU	2.4	2.4	2.0	1.8
LOC374443	2.1	2.0	1.9	1.7
MAP3K8	2.9	3.0	2.6	2.3
MBP	-2.4	-2.2	-2.3	-1.8
MCOLN2	3.8	3.9	2.9	2.8
MCTP1	2.1	1.9	1.9	1.6
MSANTD3	2.5	2.5	2.0	1.6
MYO1G	2.0	1.9	2.2	1.8
NAMPT	2.6	2.9	2.8	2.6
NBN	2.2	2.4	2.2	2.1
NFKB1	2.3	2.3	2.1	2.0
NINJ1	2.7	2.5	2.2	2.0
PDE4B	2.9	3.4	2.4	2.7
PIM2	2.8	2.9	2.3	2.1
PLAC8	2.6	2.3	2.8	2.7
PSD3	2.4	2.5	2.2	1.9
PSTPIP2	2.9	2.8	2.6	2.5
RIPK2	2.2	2.5	1.9	1.8
RNF144B	3.1	3.3	2.4	2.3
SERPINB9	2.8	2.8	2.2	2.1
SLC1A2	2.2	2.2	2.7	2.3
SLC2A6	3.7	3.7	3.2	2.9
SOCS3	2.3	2.5	2.2	2.0
SOD2	4.2	4.2	3.8	3.6

Genes	MoCT-127	MoCT-205	MoTB-127	MoTB-205
STAT4	3.8	3.8	3.4	3.2
STK26	3.8	3.7	3.0	2.7
TNFRSF21	-3.2	-2.8	-3.4	-2.6
TNFRSF4	3.6	3.6	2.8	2.6
TNIP3	2.4	2.6	2.6	2.5
TRAF1	3.1	3.1	2.5	2.3
UPB1	2.6	2.5	2.0	1.8
CCL14	2.2	2.2	1.6	
CKLF	-2.5	-2.1	-2.2	
DHRS9	-2.6	-2.2	-2.5	
GALM	-2.4	-2.4	-2.2	
HEY1	2.3	2.3	1.9	
HLA-DMB	-2.2	-2.1	-2.1	
LSS	2.2	2.1	1.9	
LTA4H	-2.2	-2.3	-2.1	
NDP	3.2	3.4	2.3	
SLC44A2	-2.7	-2.3	-2.6	
ST6GAL1	-2.3	-2.1	-2.7	
TNIP1	2.9	2.8	2.5	
TSPAN17	-2.2	-1.5	-2.0	
ZC3H12C	2.5	2.5	1.8	
CCL5	3.3	3.3		1.5
CCR7	2.8	3.1		2.8
G0S2	2.5	2.6		2.6
IL36G	3.6	3.7		2.0
PIM1	2.7	3.1		1.6
BCL11A		1.8	2.0	1.6
CASP1		1.7	1.8	1.6
CEBPA		-1.8	-1.8	-1.5
FAM49A		1.5	1.8	1.7
PFKFB3		1.6	1.7	1.7
PPARG		-1.6	-1.8	-1.6
RCBTB2		-1.7	-2.0	-1.5
SAMSN1		2.0	1.7	1.8
SLAMF7		1.9	2.0	1.7
WTAP		1.9	1.7	1.7
ZP3		1.8	2.1	2.0
29 Common genes (MoTB-127 Vs MoTB-205)				
ARHGEF6		-1.8	-1.8	
B4GALT1		1.7	1.7	
BTG3		1.8	1.5	
CORO1A		-1.5	-1.5	
DOCK10		-2.0	-2.4	
DOPEY2		-1.9	-2.0	
EVI2B		-1.6	-1.7	

Genes	MoCT-127	MoCT-205	MoTB-127	MoTB-205
EVL		-1.9	-2.4	
FBXO38		-1.9	-1.9	
GCLC		-2.1	-2.0	
GPC3		1.6	1.6	
GPR137B		1.7	1.6	
HCK		1.9	2.1	
IDH1		-1.5	-1.8	
KCTD12		-1.9	-1.5	
LOC606724		-1.7	-1.7	
MERTK		-1.6	-1.8	
MNDA		-1.9	-1.9	
PECAM1		-1.7	-1.6	
PFKFB4		-1.7	-2.2	
PLXDC2		-1.8	-1.8	
PTPRO		-1.7	-1.6	
PYCARD		-1.5	-2.0	
SMS		1.6	1.6	
SNHG15		1.8	1.8	
TEX2		-1.8	-1.5	
TIMP2		-1.7	-1.7	
TM6SF1		-1.7	-1.6	
UBASH3B		-1.9	-1.6	
52 Common genes (MoCT- 205 Vs MoCT-127)				
ACP5	-2.0	-1.8		
AVPI1	-2.1	-1.6		
CCL1	5.0	4.7		
CCL20	6.2	6.2		
CCL3	3.3	3.5		
CCL3L1	4.2	4.5		
CCL3L3	2.1	2.2		
CCL4L1	4.4	4.6		
CCL4L2	3.7	3.8		
CD40	2.1	2.5		
CD80	2.1	2.0		
CSF2	4.5	4.6		
CXCL1	4.9	5.0		
CXCL2	5.4	5.2		
CXCL8	3.6	3.6		
CYP4B1	2.4	2.3		
DPYSL3	2.4	2.2		
DUSP5	2.5	2.6		
EBI3	4.2	4.2		
EHD1	3.8	3.7		
ETS2	2.6	2.8		
FGL2	-2.5	-2.2		

Genes	MoCT-127	MoCT-205	MoTB-127	MoTB-205
ICAM1	2.0	2.2		
IDO1	2.6	3.7		
IER3	3.8	3.7		
IL1A	6.2	6.5		
IL1B	5.8	5.8		
IL23A	4.7	5.3		
IL6	5.7	6.1		
IRAK2	3.0	3.1		
IRAK3	3.0	3.1		
LAMP3	3.9	3.7		
LOC285628	2.0	2.1		
MARCKS	3.6	3.6		
MIR155HG	2.5	2.7		
MIR302C	2.1	2.1		
NFKBIA	2.2	2.3		
NFKBIZ	3.9	3.7		
PLIN2	-2.2	-2.4		
PTGS2	6.2	6.4		
RGS2	-2.2	-2.2		
RHOU	2.1	2.0		
SERPINB2	5.2	5.3		
SLAMF1	2.3	2.7		
SLC25A24	2.9	2.8		
SLC7A5	2.3	2.3		
STAT5A	2.0	2.0		
THBS1	3.0	2.5		
TNF	5.8	6.0		
TNFAIP3	2.0	2.0		
TNFAIP6	5.3	5.6		
ZC3H12A	3.0	2.7		
5 Common genes (MoCT-205 Vs MoTB-205)				
BASP1		1.7		1.8
PILRA		1.8		1.5
RGS1		-1.8		-1.5
TNFAIP2		1.7		1.6
TSPAN33		2.0		1.9
9 Common genes (MoTB--127 Vs MoTB-205)				
CCND2			-2.3	-2.1
DCSTAMP			-2.0	-1.6
EGR2			-1.6	-1.6
EMP1			-1.9	-1.6
FOS			-1.8	-1.6
MAP1LC3A			1.7	1.6
SGK223			-1.7	-1.6
SLC43A2			1.6	1.6

Genes	MoCT-127	MoCT-205	MoTB-127	MoTB-205
SPRY2			-1.9	-1.7
86 Unique genes (MoCT- 205)				
ACSL5		1.8		
AKR1B1		2.0		
ARL5B		1.7		
ASNS		1.6		
BHLHE40		1.7		
C17orf96		1.9		
CCDC109B		-1.7		
CCL2		1.5		
CCL22		2.1		
CCL23		2.0		
CCL8		2.6		
CD1D		-1.7		
CD44		1.5		
CD48		1.6		
CD82		1.6		
CENPV		-1.5		
CFLAR		1.6		
CLCF1		2.3		
CSF2RA		1.6		
CSRNP1		1.7		
CXCL10		2.4		
DNASE2		-1.6		
DUSP2		2.2		
F3		2.1		
FABP5		-1.8		
FAM129A		1.9		
FAM188A		1.7		
FAM198B		-1.5		
FCGR2A		1.6		
FPR2		1.6		
FRAT2		-1.6		
FSCN1		1.8		
FYB		-1.6		
GBP1		1.9		
GBP2		1.7		
GBP5		1.8		
GNG2		1.5		
GPR132		1.6		
HBEGF		1.6		
HELZ2		1.5		
HK1		-1.6		
HSPB1		-1.8		
IFIT2		1.6		

Genes	MoCT-127	MoCT-205	MoTB-127	MoTB-205
INSIG1		1.6		
IRF1		1.5		
ISG20		2.0		
LBR		-1.5		
LPAR6		-1.6		
MAF		-1.5		
MAFF		2.0		
MAP3K4		2.0		
MB21D2		2.1		
MSC		1.7		
MT2A		1.6		
MX1		1.5		
NCEH1		-1.7		
OSM		1.7		
P2RX7		1.7		
PDK4		-1.8		
PDSS1		1.5		
PLAUR		1.8		
PPP3CC		1.8		
PTGER4		1.5		
RAPGEF2		1.6		
REL		1.5		
RGS16		1.8		
RHOBTB3		1.5		
RNF19B		1.6		
SDC4		1.8		
SDS		-1.6		
SLC16A6		1.6		
SLC2A3		2.0		
SLC39A8		1.5		
SLC7A11		1.8		
STX11		1.5		
TGFBI		-1.6		
TMEM194A		1.7		
TNFAIP8		1.8		
TNFAIP8L3		-1.8		
TNFRSF9		1.5		
TREM2		-1.8		
TXN		1.7		
USP12		1.7		
VAV3		-1.7		
VEGFB		-1.9		
ZSWIM4		1.5		
31 Unique genes (MoTB- 127)				
ARHGEF3			-1.6	

Genes	MoCT-127	MoCT-205	MoTB-127	MoTB-205
ATP2B4			-2.2	
CHD9			-1.8	
CORO2A			-1.5	
CSF1R			-1.6	
DHRS3			-1.6	
FANCE			-1.7	
FHL1			-1.7	
GFOD1			-1.6	
HK3			-1.5	
HPCAL1			-1.7	
ITGB8			1.8	
LAD1			1.6	
METTL7B			-1.7	
NCK2			1.6	
OGFRL1			1.8	
PACSIN2			-1.5	
RAB11FIP1			-1.6	
RARRES1			1.8	
RCN1			1.5	
RDX			1.6	
SHPK			-1.6	
SLC27A3			-1.5	
SLC30A3			-2.3	
SLCO2B1			-1.8	
SLFN11			-1.5	
SORT1			-1.7	
SPRED1			-1.6	
TACSTD2			-1.6	
TBC1D10C			-1.6	
VCL			-1.6	
One Unique gene (MoTB- 205)				
GREM1				-1.9
One common gene (MoCT-127 Vs MoTB- 127)				
AGPAT9	-2.1		-2.2	
